# Spacer Fidelity Assessments of Guide RNA by Top-Down Mass Spectrometry

**DOI:** 10.1021/acscentsci.3c00289

**Published:** 2023-07-11

**Authors:** Luis A. Macias, Sara P. Garcia, Kayla M. Back, Yue Wu, G. Hall Johnson, Sekar Kathiresan, Andrew M. Bellinger, Ellen Rohde, Michael A. Freitas, James A. Madsen

**Affiliations:** † Verve Therapeutics, 201 Brookline Avenue, Suite 601, Boston, Massachusetts 02215, United States; ‡ MassMatrix, Inc., 600 Teteridge Road, Columbus, Ohio 43214, United States; § The Ohio State University, 281 West Lane Avenue, Columbus, Ohio 43210, United States

## Abstract

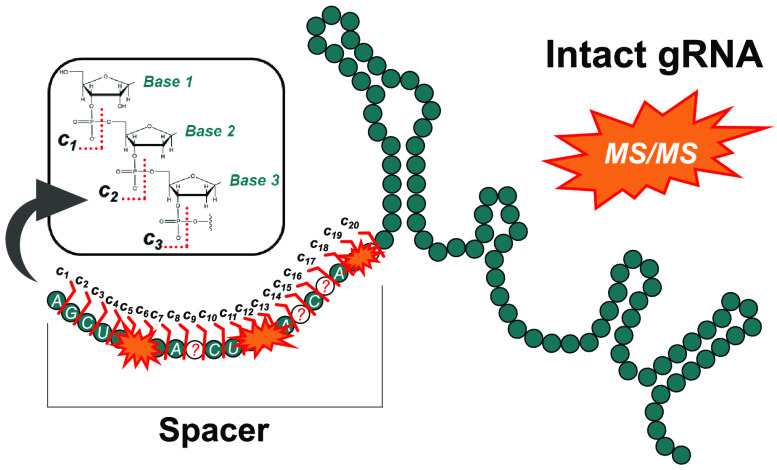

The advancement of CRISPR-based gene editing tools into biotherapeutics offers the potential for cures to genetic disorders and for new treatment paradigms for even common diseases. Arguably, the most important component of a CRISPR-based medicine is the guide RNA, which is generally large (>100-mer) synthetic RNA composed of a “tracr” and “spacer” region, the latter of which dictates the on-target editing site as well as potential undesired off-target edits. Aiming to advance contemporary capabilities for gRNA characterization to ensure the spacer region is of high fidelity, top-down mass spectrometry was herein implemented to provide direct and quantitative assessments of highly modified gRNA. In addition to sequencing the spacer region and pinpointing modifications, top-down mass spectra were utilized to quantify single-base spacer substitution impurities down to <1% and to decipher highly dissimilar spacers. To accomplish these results in an automated fashion, we devised custom software capable of sequencing and quantifying impurities in gRNA spacers. Notably, we developed automated tools that enabled the quantification of single-base substitutions, including advanced isotopic pattern matching for C > U and U > C substitutions, and created a *de novo* sequencing strategy to facilitate the identification and quantification of gRNA impurities with highly dissimilar spacer regions.

## Abstract 2

A mass spectrometry strategy for assessing the sequence fidelity of the critical gene-edit directing spacer region of CRISPR guide RNA was developed for sequence impurity discovery and quantitation.

## Introduction

CRIPSR-based gene editing has the potential to transform modern medicine by offering *in vivo* and *ex vivo* treatments and cures for genetic diseases. Recently, we have shown successful *in vivo* proof-of-concept in nonhuman primates^1,2^ for potential single-course gene editing treatments for atherosclerotic cardiovascular disease,^1^ the worldwide leading cause of death,^3^ and other groups have demonstrated proof-of-concept as well in humans treating transthyretin amyloidosis.^4^ Such *in vivo* CRISPR-based gene editing medicines are commonly comprised of guide RNA (gRNA) that functions to target the gene editing enzymes to the desired genomic location and messenger RNA (mRNA) that encodes the gene editing enzymes (*e.g.*, CRISPR Cas nucleases,^5−7^ CRISPR base editors,^8−12^ and CRISPR prime editors^13^), both of which are encapsulated in lipid nanoparticles.

**Figure 1:**
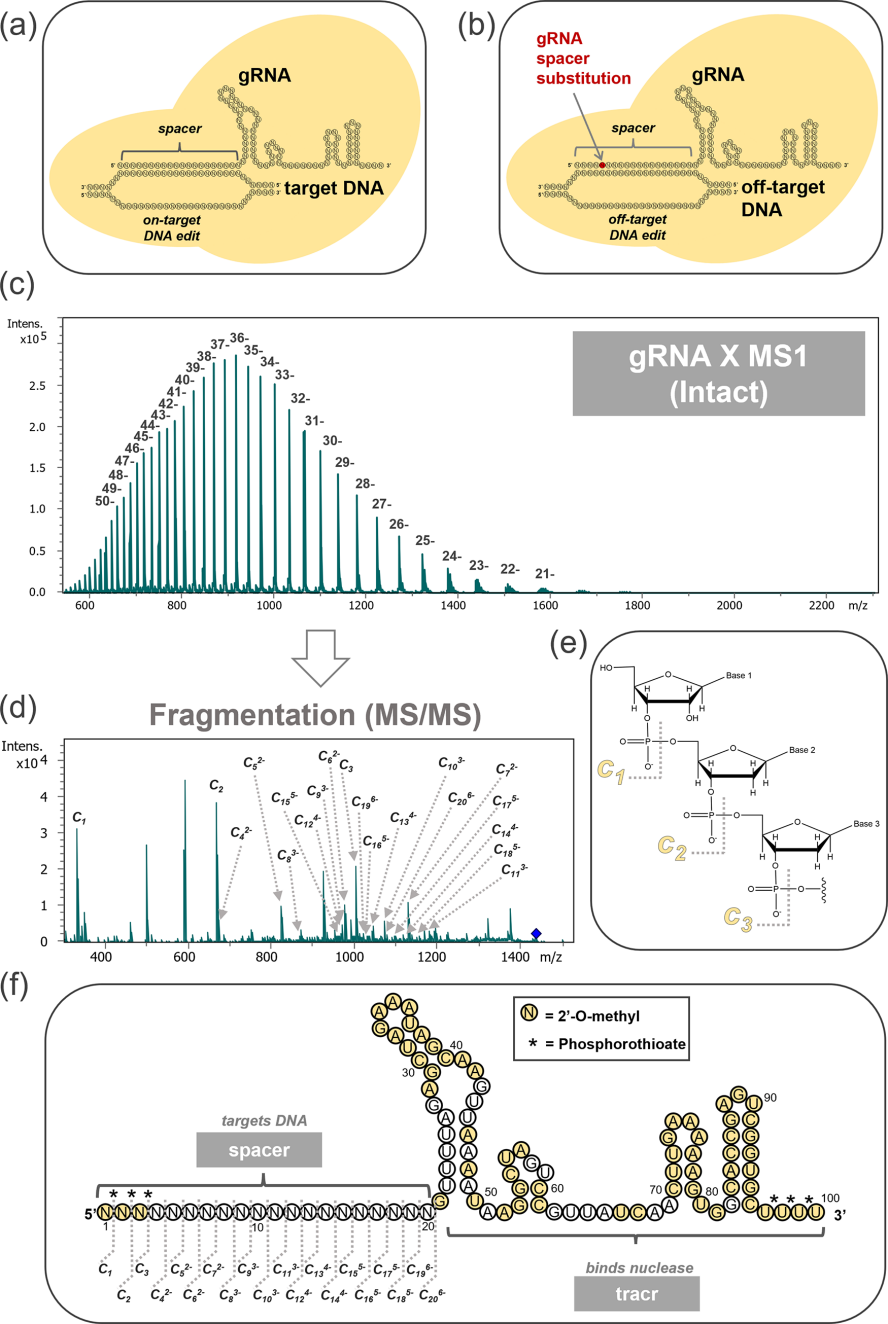
Overview of the potential off-target consequences of an unintended gRNA spacer substitution: (a) desired on-target DNA edit and (b) potential off-target DNA edit caused by unintended spacer substitution. The yellow space represents a CRISPR nuclease/nickase. Top-down gRNA spacer sequencing: (c) an example full mass spectrum of gRNA X, (d) an example top-down MS/MS spectrum of gRNA X, (e) *c*-type product ion structure/nomenclature, and (f) MS/MS product ions matched to an example gRNA X spacer region. The letter “N” denotes any RNA nucleotide—A, C, G, or U.

Given that the gRNA dictates on- and off-target specificities for CRISPR-based medicines, accurately characterizing the fidelity and purity of such guides is commensurately important. Typically, single guide RNAs are created synthetically and, in the case of Cas9 gRNA, are made up of approximately 100 nucleotides divided into two major parts, the “tracr” region, which binds to the nuclease/nickase, and importantly the spacer region (*i.e.*, the first 20 nucleotides from the 5′-end), which targets the intended DNA editing location (protospacer). Common guide designs may comprise a nucleotide sequence ranging from 70 to 200 nucleotides in length and may include various chemical modifications directed to enhance functionality such as improvements to *in vivo* stability or to decrease off-target editing. These modifications may include methylations, phosphorothioate linkages, DNA/RNA hybrids, and other chemical alterations at specific nucleotide locations.^14−17^ Various chemical modifications may be included at specific nucleotide locations in the tracr or spacer region. Conventional chemical synthesis of such long oligonucleotides is error-prone, and together with purification challenges, it is not uncommon for gRNA drug substances to have a notable lack of fidelity and to have a variety of types of impurities. These issues tend to increase with the length of the gRNA or the amount and nature of the chemical modifications. As illustrated in Figure 1a,b, the lack of fidelity in the spacer region is particularly concerning because of the potential to cause edits to unintended genes or locations. As an example of an off-target effect due to a single-base substitution, the VEGFA site 3 guide (GGTGAGTGAGTGTGTGCGTG) has a well-known off-target site with a single sequence mismatch (GGTGAGTGAGTGTGTGTGTG), which has been identified by methods such as GUIDE-seq^18^ and ONE-seq.^19^

The characterization of gRNA, including an in-depth assessment of spacer fidelity, can provide critical insight into the quality and performance of CRISPR-based medicine. Established sequencing methods, such as next-generation sequencing (NGS),^20^ PacBio,^21^ and Oxford Nanopore technologies,^22^ are insufficient to directly, unambiguously, and quantitatively characterize gRNA, especially long and/or highly modified guides. NGS and PacBio, for example, are indirect measurements that rely on reverse transcriptase, which has the potential to introduce bias or other inaccuracies. These methodologies also have challenges with assessing RNA with many chemically modified nucleotides.^23,24^ Nanopore, on the other hand, while more direct has often been found to be inaccurate due to the lack of base calling routines that can account for modifications.^22^ Mass spectrometry (MS) has successfully been employed to characterize both RNA and DNA but has generally proved best suited for smaller oligonucleotides.^25−40^ Recently, MS-based methodologies have been applied to larger RNA; however, such techniques have most often relied on digesting the RNA first into smaller pieces prior to mass spectrometry analysis, also known as bottom-up sequencing.^41−45^ These workflows often lead to small fragments that lack sequence specificity and have the potential to introduce sample preparation-induced artifacts.^46,47^ Top-down or direct sequencing of RNA by mass spectrometry has been applied to modified tRNA,^48,49^ but this RNA type is considerably shorter and less modified than CRISPR gRNA. Furthermore, sequence fidelity assessed by highly sensitive and quantitative methodologies was not evaluated. It is therefore here recognized that novel tools are needed.

The technology described herein is utilized for an in-depth assessment of gRNA spacer fidelity. The methodology involves directly fragmenting intact gRNA inside a mass spectrometer to preferentially generate *c*-type ions that unambiguously cover the entire spacer sequence. This method can site-specifically identify both sequence and chemical impurities and quantify spacer base substitutions down to <1%. Furthermore, we developed automated data analysis software capable of interpreting the top-down spectra with advanced identification scoring, quantitating unknown spacer sequence impurities including isotopic matching for low-level C > U and U > C substitutions, and *de novo* sequencing of guide impurities with markedly different spacer sequences.

## Experimental Procedures

### Materials

All gRNA was obtained from BioSpring (Frankfurt, Germany) and purified using a process optimized for producing a full-length product. The gRNA sequences utilized in this study can be found in Table 1. Spacer sequences for these guides are also shown in Table 2. All other reagents and chemicals were obtained from Thermo Fisher Scientific (Waltham, MA).

**Table 1:** All gRNA Sequences Utilized in This Study^a^

name	sequence	average mass (Da)
gRNA X	C(ms)A(ms)G(ms)GUUCCAUGGGAUGCUCUG(m)UUUUAGA(m)G(m)C(m)U(m)A(m)G(m)A(m)A(m)A(m)U(m)A(m)G(m)C(m)A(m)A(m)GUUA(m)AA(m)AU(m)AA(m)G(m)G(m)C(m)U(m)A(m)GUC(m)C(m)GUUAU(m)C(m)AAC(m)U(m)U(m)G(m)A(m)A(m)A(m)A(m)A(m)G(m)U(m)G(m)GC(m)A(m)C(m)C(m)G(m)A(m)G(m)U(m)C(m)G(m)G(m)U(m)G(m)C(m)U(ms)U(ms)U(ms)U(m)	33,148.39
gRNA XA	C(ms)U(ms)G(ms)GUUCCAUGGGAUGCUCUG(m)UUUUAGA(m)G(m)C(m)U(m)A(m)G(m)A(m)A(m)A(m)U(m)A(m)G(m)C(m)A(m)A(m)GUUA(m)AA(m)AU(m)AA(m)G(m)G(m)C(m)U(m)A(m)GUC(m)C(m)GUUAU(m)C(m)AAC(m)U(m)U(m)G(m)A(m)A(m)A(m)A(m)A(m)G(m)U(m)G(m)GC(m)A(m)C(m)C(m)G(m)A(m)G(m)U(m)C(m)G(m)G(m)U(m)G(m)C(m)U(ms)U(ms)U(ms)U(m)	33,125.35
gRNA XB	C(ms)A(ms)G(ms)GUUCCAUAGGAUGCUCUG(m)UUUUAGA(m)G(m)C(m)U(m)A(m)G(m)A(m)A(m)A(m)U(m)A(m)G(m)C(m)A(m)A(m)GUUA(m)AA(m)AU(m)AA(m)G(m)G(m)C(m)U(m)A(m)GUC(m)C(m)GUUAU(m)C(m)AAC(m)U(m)U(m)G(m)A(m)A(m)A(m)A(m)A(m)G(m)U(m)G(m)GC(m)A(m)C(m)C(m)G(m)A(m)G(m)U(m)C(m)G(m)G(m)U(m)G(m)C(m)U(ms)U(ms)U(ms)U(m)	33,132.39
gRNA XC	C(ms)A(ms)G(ms)GUUCCAUGGGAUGCUCGG(m)UUUUAGA(m)G(m)C(m)U(m)A(m)G(m)A(m)A(m)A(m)U(m)A(m)G(m)C(m)A(m)A(m)GUUA(m)AA(m)AU(m)AA(m)G(m)G(m)C(m)U(m)A(m)GUC(m)C(m)GUUAU(m)C(m)AAC(m)U(m)U(m)G(m)A(m)A(m)A(m)A(m)A(m)G(m)U(m)G(m)GC(m)A(m)C(m)C(m)G(m)A(m)G(m)U(m)C(m)G(m)G(m)U(m)G(m)C(m)U(ms)U(ms)U(ms)U(m)	33,187.43
gRNA XD	C(ms)A(ms)G(ms)GCUCCAUGGGAUGCUCUG(m)UUUUAGA(m)G(m)C(m)U(m)A(m)G(m)A(m)A(m)A(m)U(m)A(m)G(m)C(m)A(m)A(m)GUUA(m)AA(m)AU(m)AA(m)G(m)G(m)C(m)U(m)A(m)GUC(m)C(m)GUUAU(m)C(m)AAC(m)U(m)U(m)G(m)A(m)A(m)A(m)A(m)A(m)G(m)U(m)G(m)GC(m)A(m)C(m)C(m)G(m)A(m)G(m)U(m)C(m)G(m)G(m)U(m)G(m)C(m)U(ms)U(ms)U(ms)U(m)	33,147.41
gRNA Y	C(ms)C(ms)C(ms)GCUCGUUAACGCAACGGG(m)UUUUAGA(m)G(m)C(m)U(m)A(m)GA(m)A(m)A(m)U(m)A(m)G(m)C(m)A(m)A(m)GUUA(m)AA(m)AU(m)AA(m)G(m)G(m)C(m)U(m)A(m)GUC(m)C(m)GUUAU(m)C(m)AAC(m)U(m)U(m)GA(m)A(m)A(m)A(m)A(m)G(m)U(m)G(m)GC(m)A(m)C(m)C(m)G(m)A(m)G(m)U(m)C(m)G(m)G(m)U(m)G(m)C(m)U(ms)U(ms)U(ms)U(m)	33,101.39

a The sequence syntax used in the table is based on algorithms used for mass spectrometry data representation and analysis. N and N(m) respectively indicate a ribonucleotide and a 2′-O-methylribonucleotide. N(ms) denotes a phosphorothioate backbone at the 3′-end of the preceding 2′-O-methylribonucleotide.

**Table 2:** Spacer Sequences (the First 20 Nucleotides on the 5′-End) for the gRNA Sequences Shown in Table 1^a^

name	spacer
gRNA X	C(ms)A(ms)G(ms)GUUCCAUGGGAUGCUCU
gRNA XA	C(ms)U(ms)G(ms)GUUCCAUGGGAUGCUCU
gRNA XB	C(ms)A(ms)G(ms)GUUCCAUAGGAUGCUCU
gRNA XC	C(ms)A(ms)G(ms)GUUCCAUGGGAUGCUCG
gRNA XD	C(ms)A(ms)G(ms)GCUCCAUGGGAUGCUCU
gRNA Y	C(ms)C(ms)C(ms)GCUCGUUAACGCAACGG

a The tracr regions (nucleotides 21–100 in the Table 1 sequences) are highly similar for each sequence. The sequence syntax used in the table is based on algorithms used for mass spectrometry data representation and analysis. N(ms) denotes a phosphorothioate backbone at the 3′-end of the preceding 2′-O-methylribonucleotide.

### Liquid Chromatography-Tandem Mass Spectrometry (LC-MS/MS)

All gRNA samples were prepared at 10 mg/mL in water and injected (20 μL) on a Thermo Accucore, C30, 2.6 μM, 2.1 mm × 250 mm column (Waltham, MA) using a Waters H-Class Quaternary UPLC (Milford, MA) directly coupled to a Bruker Daltonics maXis II ultrahigh resolution QTOF mass spectrometer (Billerica, MA). Mobile phase A (MPA) was 100 mM of 1,1,1,3,3,3-hexafluoro-2-propanol (HFIP), 16.3 mM of triethylamine (TEA), and 1% methanol; mobile phase B (MPB) was 50% methanol and 50% acetonitrile; and mobile phase C (MPC) was 50% methanol and 0.1% formic acid. MPC was used as a wash solution at the end of the gradient to reduce metal adducts in the LC-MS system.^50^ The column was run at an ambient temperature with a flow rate of 250 μL/min and a linear gradient of 95:5% MPA/MPB to 50:50% MPA/MPB over 5 min followed by 3 min of MPC and 4 min of equilibration. The Bruker maXis II was set to negative polarity, a spectral rate of 1.00 Hz, a source capillary of 4500 V, a source dry gas of 8.0 L/min, a source dry temperature of 220 °C, an MRM mass of 1439.5 *m*/*z*, an MRM width of 200.0 *m*/*z*, and an MRM collision energy of 34.0 eV.

### Data Analysis

Top-down spectra were averaged over the elution period of gRNA during LC-MS/MS. For manual MS/MS spectral interpretation, *c*-type product ions from the gRNA spacer were identified in the averaged MS/MS spectra by matching the experimental and theoretical monoisotopic *m*/*z* values to less than 0.01 *m*/*z* as well as ensuring the correct charge state via a discernible isotopic profile. Automated data analysis was conducted by converting all LC-MS/MS experiment files into the mzML file format using MSConvert from ProteoWizard.^51^ Data were then analyzed with mmOligo software (MassMatrix, Inc.) and custom Python scripts using the pyOpenMS and SciPy packages. Workflow *in silico* involved averaging spectra across the gRNA elution period, followed by centroiding, removal of signal counts <500 to reduce noise, and removal of signals >1310 *m*/*z* to eliminate predominant uninformative signals due to surviving precursor ions. This *m*/*z* cutoff was selected to mitigate the effects of increased background noise above this *m*/*z* that resulted from the large isolation window (200 *m*/*z*) applied. The resulting MS/MS spectra were then searched for *c-*fragment ions that would be produced for fragments *c*_1_–*c*_20_ in the 1- to 7- charge states for a gRNA with a given sequence. To examine sequence coverage across the entire 100-mer, the fragment search was extended to cover fragment ions *c*_1_–*c*_99_ and *y*_1_–*y*_99_ in the 1- to 25- charge states. All fragment matches constituted a mass error of <0.01 *m*/*z*, matching of at least 2 consecutive isotopes, and a cosine score of >0.80 (eq 1). Theoretical fragment ion distributions were predicted from the chemical composition and, in the case of C/U distinctions, fitted to the observed isotopic distribution as a mixture of both C and U compositions by a non-negative least-squares (nnls) approach and subsequent minimizing of the cosine distance between the initial nnls estimate and the observed distribution. For single-base substitution discovery experiments, scores were calculated as a sum of fragment intensities (represented by the signal count for the most abundant ion in the theoretical isotopic distribution) multiplied by the square of the cosine score (to penalize poor matches) for *c-*fragment ions between *c*_*n*_ and *c*_*m*_, where *n* is the base substitution site and *m* is the final position in the spacer sequence. Scores were then weighed based on the square of the percent sequence coverage from position *n* to *m* to penalize poor coverage (eq 2). For *de novo* sequencing, scores were equal to the sum intensity of all matched fragments multiplied by the square of their respective cosine scores (eq 3). When weighed by coverage, final scores were simply multiplied by the square of the percent sequence coverage (eq 4).
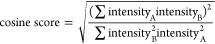
1
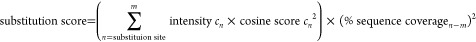
2
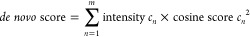
3
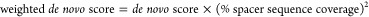
4

### NGS of gRNA

Reverse transcription of gRNA (100 ng/μL) was performed on a Bio-Rad T100 (Hercules, CA) using a direct primer to the 3′-end and Mastermix from the Takara SMARTer smRNA-Seq for the Illumina kit. Libraries were constructed from the resulting cDNA via polymerase chain reaction and incorporated with Illumina P5 and P7 adapters as well as Illumina TruSeq CD index sequences using the Takara SMARTer smRNA-Seq for the Illumina kit. Subsequent purification with NucleoSpin Gel and PCR Clean-Up kit was performed, followed by quantitation on a Qubit 4 Fluorometer (Waltham, MA) using a Qubit 1× dsDNA High Sensitivity Assay kit. Fragment size was confirmed on a 4200 TapeStation System (Santa Clara, CA) using a D1000 ScreenTape Assay. Libraries were then pooled and prepared for sequencing at 12 pM with 10% PhiX, prior to loading onto a MiSeq V2 Reagent Cartridge. Library sequencing was performed on an Illumina MiSeq System (San Diego, CA) using a Nano Flow Cell with two 120-run cycles.

Reads were aligned to the respective guide sequence with CRISPResso2 v2.0.31^52^ using parameters “--max_paired_end_reads_overlap 160 --default_min_aln_score 60 --min_bp_quality_or_N 30 --exclude_bp_from_left 0 --exclude_bp_from_right 0 −e quantification_window_center -50 --quantification_window_size 50 --base_editor_output --conversion_nuc_from A --conversion_nuc_to G --plot_window_size 50 --min_frequency_alleles_around_cut_to_plot 0.1 --max_rows_alleles_around_cut_to_plot 200”.

## Results and Discussion

### Evaluation of Sensitivity for Top-Down Mass Spectrometry of Highly Modified gRNA

We first developed a mass spectrometry-based workflow focused on assessing spacer fidelity with a direct and sensitive measurement for a large, highly modified gRNA. An overview of the direct, top-down methodology is illustrated in Figure 1c–f, exhibiting a representative analysis of gRNA X. In this strategy, ion-pairing reverse-phase high-performance liquid chromatography (IP-RP) is first implemented to introduce gRNA into a high-resolution mass spectrometer (QTOF) optimized for the maximum ion signal. A representative full mass spectrum for intact gRNA X is depicted in Figure 1c. The intact gRNA precursor ions were then isolated in a broad isolation window (200 *m*/*z*) set to capture lower charge states (26- to 20-) and fragmented by tandem mass spectrometry (MS/MS), a process that accelerates the ions into a bath gas to induce vibrational heating and subsequent dissociation along the RNA backbone. The wide isolation width that encompassed many lower-charged precursor ions was selected, as it yielded the best combination of a rich fragmentation behavior and high product ion signals for desired sequence fragments, as depicted in the top-down MS/MS spectrum of gRNA X in Figure 1d. Upon dissociation of intact gRNA, fragmentation occurs readily at the 5′- and 3′-ends, advantageously affording specific and sensitive assessment of the 5′-end spacer section via the formation of *c*-type ions (Figure 1e). This phenomenon provides increased sensitivity for the spacer region since the product ion signal is funnled into fewer ion channels. A fragment ion series spanning *c*_1_–*c*_20_, as annotated in Figure 1d, yielded full coverage of the vital spacer sequence (Figure 1f). Complete sequencing by *c*-type product ions of the entire spacer region (first 20 nucleotides from the 5′-end) was achieved for all of the gRNA sequences (see Table 1) utilized herein. In addition, the technology can site-specifically identify and quantify both sequence and chemical modifications as long as a given impurity has a different mass. As shown in Figure 1f, sequencing of the gRNA X spacer region was undeterred by chemical modifications and even enabled the detection and precise localization of 2′-O-methylation and phosphorothioate modifications to the first three nucleotides.

**Figure 2:**
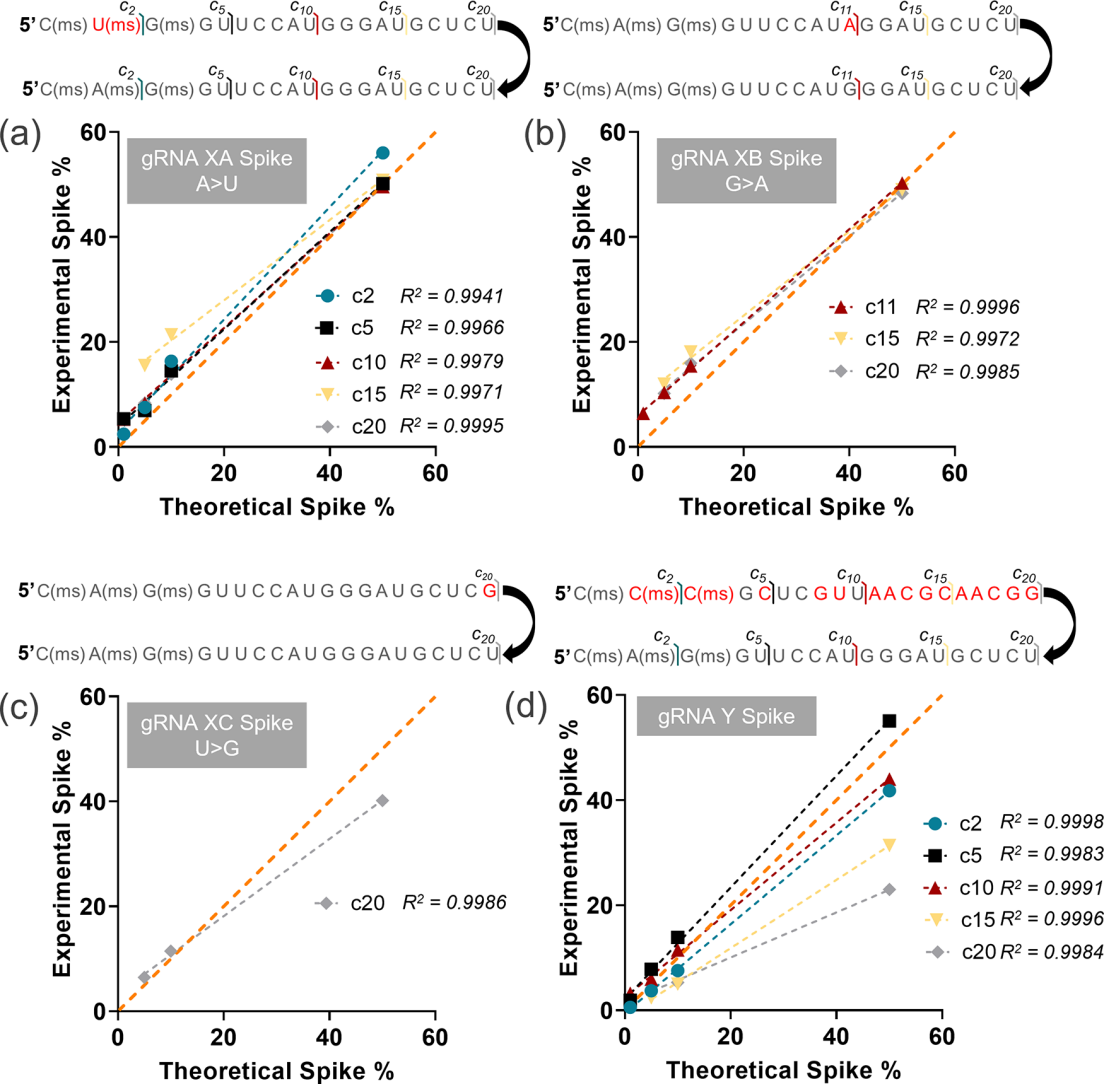
Top-down gRNA spacer sequence quantitation: gRNA X was spiked with 1, 5, 10, and 50% total concentrations of (a) gRNA XA differing by an A > U substitution, (b) gRNA XB differing by a G > A substitution, (c) gRNA XC differing by a U > G substitution, and (d) gRNA Y featuring multiple base substitutions. Concentrations of the spiked-in gRNA, as determined by selected fragment ion abundances, were plotted as a function of the in-solution concentration. Lines of best fit produced by linear regression are shown in dashed lines for each fragment ion. *R*^2^ values determined by linear regression are shown for each fragment ion. Orange dashed lines represent the line of identity, *x = y*. Spacer nucleotides that differ between the two gRNA are denoted in red font. N(ms) denotes a phosphorothioate backbone at the 3′-end of the preceding 2′-O-methylribonucleotide.

To further assess the sensitivity of the MS/MS sequencing method and demonstrate practical utility for impurity analysis, mixtures of gRNAs with different spacer sequences were similarly evaluated at multiple concentrations. Three variants of gRNA X (denoted in Table 1 as gRNA XA, XB, and XC), with a single-base substitution at a different position along the spacer sequence, were each individually spiked into gRNA X at 1.0, 5.0, 10, and 50% total gRNA concentrations (Figure 2). These 4 data points were selected to evaluate the ability of this strategy to inform the presence of low-abundance impurities that may occur during gRNA synthesis. Each mixture was subsequently analyzed by MS/MS to produce fragment ions for the gRNA spacer and the spiked-in variant within the same spectrum, permitting relative quantitation through the comparison of fragment ion intensities. For the variant gRNA XA, featuring an A > U base substitution at position 2 from the 5′-end, the abundance of *c*_2_, *c*_5_, *c*_10_, *c*_15_, and *c*_20_ fragment ions for the substituted sequence was calculated as a percentage of the total *c*-ion abundance at each position, *i.e.*, the sum of the variant XA *c*_*n*_ ion abundance and gRNA X *c*_*n*_ ion abundance, and plotted as a function of the percent total gRNA concentration in solution (Figure 2a) to track changes in fragment abundance throughout the spacer. Overall, a positive linear correlation was observed for all four fragment ions with sensitivity down to 1% for *c*_2_, *c*_5_, and *c*_10_ and 5% for the larger *c*_15_ and *c*_20_ fragment ions, demonstrating that this approach is sufficiently sensitive for the detection and relative quantitation of low-abundance spacer sequence impurities. Sensitivity at a 1% total concentration for gRNA XA is further demonstrated by Figure S1 where spectra for gRNA X with 0 and 1% gRNA XA are compared. In these spectra, the gRNA XA *c*_2_ fragment monoisotopic peak (theoretical *m*/*z* = 670.0444) is present at a significantly higher intensity compared to the noise level observed in the 0% spectrum. A similar trend was observed for the variant gRNA XB, possessing a G > A substitution at position 11 from the 5′-end, where base-substituted *c*_11_ was detected even at a 1% spike-in concentration and at 5% for *c*_15_ and *c*_20_ (Figure 2b). Smaller fragments, such as *c*_5_ for XB, were excluded from this analysis, as the mass of the variant XB *c*_5_ fragment would be equal to the mass of the gRNA X *c*_5_ fragment and thus indistinguishable by the MS analysis. Although this limitation produced no major effect on the analysis of gRNA XB, the impact became more pronounced when the substitution occurs further from the 5′-end, as demonstrated for gRNA XC (featuring U > G at position 20) where only *c*_20_ is unique to this sequence (Figure 2c), leading to a sensitivity down to a 5% spike-in concentration, whereas 1% was achieved for all other variants with base substitutions closer to the 5′-end. The sensitivity of the methodology, essentially, decreases as the base substitution position increases. Furthermore, although some deviations from linearity were observed in each spike-in experiment (Figure 2a–c), these deviations do not dramatically inhibit the characterization of spacer fidelity. Performing a linear regression for each fragment ion evaluated for each gRNA mixture revealed a high *R*^2^ value of >0.99, indicating that these deviations do not obfuscate the overall linear correlation between the measured gRNA percent concentration and the actual gRNA percent concentration. Divergence from linearity is attributed to different responses from the distinct gRNA spacer sequences that would arise from variation in fragmentation efficiency due to changes in sequence or concomitant changes in the gas-phase structure.

Remarkably, relative quantitation and detection of gRNA with distinct spacers remained possible, even for highly dissimilar spacer sequences. As demonstrated in Figure 2d, gRNA Y, differing from gRNA X at 15 base positions (shown in red font in Figure 2d), was spiked into gRNA X at 1.0, 5.0, 10, and 50% total gRNA concentrations and assessed by MS/MS. As with the single-substitution variants, the sensitivity of the method was demonstrated down to 1% for smaller product ions (*c*_2_, *c*_5_, *c*_10_) and 5% for larger product ions (*c*_15_, *c*_20_) via comparison of fragment ion intensities for the respective sequences. Deviations from the line of identity in Figure 2d are more pronounced than for the single-substitute variants (Figure 2a–c), particularly for larger fragments *c*_15_ and *c*_20_ where the sequences are most dissimilar between gRNA X and gRNA Y. These distortions also likely arise from sequence-induced changes in fragmentation efficiency and response. Analogous to results for the single-substitution variants, the assessment of spacer fidelity and increases in gRNA X concentration remained possible despite potential differences in the response between the two guides. Altogether, these results exemplify the power of top-down MS/MS analyses to detect impurities present on the critical spacer region of gRNA while retaining the ability to perform relative quantitation.

Further contextualizing gRNA sequencing via top-down mass spectrometry, all gRNA sequences were also analyzed by NGS, the gold standard in DNA and RNA sequencing, to generate a comparative data set and evaluate the advantages of each approach. Impressively, NGS is capable of perfectly sequencing the entire 100-mer (Figure S2a), while the current MS/MS methodology is more limited in this metric beyond the spacer region (see the Sequence Coverage beyond the gRNA Spacer section). However, in order to perform NGS on RNA and sequence the nucleotides, the RNA must first be reverse-transcribed into DNA to undergo polymerase chain reaction (PCR), resulting in a loss of information that, fortuitously, may be recovered by the use of MS/MS. The most striking disadvantage is the loss of RNA modification information in NGS analyses; as discussed briefly above, the presence of modifications did not inhibit sequencing by MS/MS resulting in complete characterization and localization of the 2′-O-methylation and phosphorothioate modifications present on the first three nucleotides of the spacer sequence. Magnifying the capabilities of this attribute, MS/MS for RNA modification characterization is further developed and discussed in the *De Novo* RNA Modification Assignment and Localization section to capitalize on aspects of MS/MS that are not easily matched by NGS. Secondly, RT-PCR can potentially introduce biases that alter library yield and negatively impact quantitative metrics. As seen in Figure S2b and Table S1, variability was observed even for sequences that differ by only a single-base substitution, exemplifying the effects of potential bias. As expected, lower library yields correlated with a subsequent decrease in the number of reads that matched the reference sequence (Figure S2a). Such biases limit the use of NGS for the quantitative analysis of impurities, as small changes in the impurity nucleotide sequences may pose drastic changes to the library yield and number of matched reads. In contrast, MS/MS displayed adequate quantitative merits, even for mixtures of these gRNA. Although the biases found in NGS did not affect the sequencing of the gRNA, this impact on library yield can affect the ability to quantify sequence impurities identified via NGS. However, careful application of MS/MS to supplement NGS analyses can serve to redress these issues; accordingly, quantitative aspects of the MS/MS strategy remain a focus for the remainder of the discussion. Altogether, tandem mass spectrometry is positioned to become an excellent complement to the already powerful oligonucleotide sequencing strategy of NGS.

### Development of Automated Data Analysis for Characterization of gRNA Spacer Fidelity

Although MS/MS enables powerful characterization of gRNA spacer fidelity, adequately capitalizing on such capabilities demands workflows that circumvent the limitations of manual spectral interpretation. In contrast to mature methodologies, top-down mass spectrometry and oligonucleotide mass spectrometry currently lack widespread availability of dedicated data analysis software packages that have allowed extensive adoption of mass spectrometry assays for routine high-throughput studies in now-ubiquitous applications, such as bottom-up proteomics and metabolomics. To address this shortcoming, we developed software, such as mmOligo and additional Python scripts, to confidently interpret top-down MS/MS spectra and assign *c-*ion fragments spanning gRNA spacer sequences. Details regarding the automated assignment of gRNA fragment ions are described in the Experimental Procedures section. High mass accuracy and isotopic fidelity were ensured by establishing a low mass error cutoff appropriate for the instrumental accuracy (<0.01 *m*/*z*) and by measuring a cosine similarity score between the observed isotopic distribution and theoretical distribution.

To evaluate the accuracy of fragment assignments via this approach, an estimate of the false discovery rate (FDR) of oligonucleotide sequence fragments was performed by adopting a strategy previously described to evaluate the FDR of intact protein sequence fragments. Estimating FDR by this method entailed generating decoy fragment masses by offsetting the fragment ion *m*/*z* values from −*X* to +*X* ppm in incremental steps; all assignments made for the offset fragment lists were treated as random hits, while all assignments made for the unmodified fragment mass list were treated as target hits.^55,56^ The number of random hits for each offset mass list divided by the number of target hits estimates the number of false positives per all positive assignments, providing an approximation of the FDR. Applying this strategy to evaluate the FDR of gRNA top-down fragment assignments, all theoretical *c*-fragment *m*/*z* values for gRNA X spacer were offset from −1000 to +1000 ppm in 50 ppm steps and searched for in the corresponding top-down spectrum; the total number of hits for each offset fragment list is denoted in Table S2 and Figure S3a. In an initial analysis performed without applying a cosine score cutoff, the average FDR for the offset data sets was determined to be 13.4%, corresponding to a median of 4 random fragment assignments per search. To identify an appropriate cosine score cutoff, the histogram in Figure S3b was constructed to visualize the distributions of cosine scores for random and target hits (*i.e.*, fragments identified using a theoretical fragment list with no ppm offset), revealing a clear and tight distribution for the target hits approaching a cosine score of 1, which contrasts with the broad and random distribution observed for the random hits. Applying a cosine score cutoff of 0.80 consequently enables the assignment of all gRNA X sequence fragments while excluding a majority of false assignments. Recalculating FDR with a cosine score threshold of 0.80 dramatically reduces FDR for each offset fragment list (Figure S3a) and the median number of random fragment assignments to 3.7% and 1, respectively. Although the FDR could be reduced further by increasing the cosine score threshold, applying a higher threshold would also exclude fragment ions that were confidently identified by manual analysis. For example, one *c*_15_^5–^ product ion had a cosine score of 0.84 due to mild overlap with the isotopic distribution of *c*_9_^3–^ (Figure S4). Because a major goal of this automated strategy is the discovery of low-abundance spacer sequence impurities for synthetic RNA (impurities that would likely produce fragment ions obfuscated by more abundant fragments originating from the main RNA product), a score threshold of 0.80, which captures all fragments identified via manual interpretation, even at the cost of a slightly higher FDR, was deemed appropriate for this workflow.

Applying the resulting characterization power to address sequence confirmation, the automated assignment of RNA sequence fragments was shown to achieve complete coverage for the spacer regions of all gRNA sequences assessed herein, as shown in Figure S5. Fragment ions identified in the MS/MS spectra of each gRNA are tabulated in Tables S3–S8. Generally, 28–31 *c*-type fragments, spanning *c*_1_–*c*_20_ in multiple charge states up to the 6- charge state, were detected for each of the six gRNA spacer sequences with high mass accuracy (<0.01 *m*/*z* mass error) and high isotopic fidelity (cosine score > 0.80). Ultimately, the automated assignment of gRNA sequence fragments by tailored software, such as mmOligo, empowered rapid, confident, and complete sequence confirmation of multiple gRNA spacers while circumventing the necessity for tedious manual spectral interpretation and setting a foundation for additional developments in spacer fidelity assessments.

### Sequence Coverage beyond the gRNA Spacer

Although the characterization of the gRNA spacer is key for the proper function of the biomolecule and remains the ultimate goal of the described methodology, the characterization of the entire 100-mer is still remarkably possible with the same strategy discussed thus far. To demonstrate, spectra collected for each of the gRNA were re-evaluated to search for *y-*ions and for product ions from across the entire sequence, essentially searching for *c*_1_–*c*_99_ and *y*_1_–*y*_99_. Identified fragment ions are listed in Tables S3–S8. Results for gRNA X are displayed in Figure S6, showing a coverage map (Figure S6a) along with an annotated spectrum (Figure S6b). Additional coverage maps for gRNA XA, gRNA XB, gRNA XC, gRNA XD, and gRNA Y are provided in Figure S7. Overall, in addition to complete coverage of the spacer, good sequence coverage ranging from 58 to 68%, with a median of 65%, was achieved for the complete gRNA sequences. Because each of these gRNA features a highly similar tracr region, fragmentation trends beyond the spacer were highly consistent. For at least half of the gRNA sequences, *c*-type fragments *c*_22_–*c*_28_, *c*_*32*_, *c*_*43*_, and *c*_*47*_ were identified consistently, in addition to other fragments that occurred more sporadically across the different sequences. Regarding *y-*type fragments, smaller mass ions including *y*_2_, *y*_3_, *y*_5_, and *y*_6_ were found for all sequences. Furthermore, good coverage, although incomplete, was observed from *y*_2_–*y*_41_, covering about half of the tracr region with additional coverage provided by fragments occurring nonsequentially at different positions across the different gRNA sequences. Importantly, all of the identified *c*- and *y-*type fragment ions retained the 2′-O-methylation and phosphorothioate modifications present throughout the sequence, signaling not only that MS/MS gRNA sequencing is unperturbed by RNA modifications but also that MS/MS can be empowered to identify and localize such modifications; this latter feature is discussed extensively in the *De Novo* RNA Modification Assignment and Localization section.

Although both *c-*type and *y-*type fragments could be identified, “golden pairs” of *c-* and *y*-ions originating from the same fragmentation site were scarcely observed and were found only infrequently throughout the gRNA sequences. Instead, *c-*type fragments were predominantly observed near the 5′-end, while *y-*type fragments strongly trended near the 3′-end, with low overlap between the two fragment types. This trend aligns with previous observations noted for top-down analyses of intact proteins, where secondary fragmentation of larger fragment ions or spectral congestion of the larger ions is proposed to favor the formation and detection of smaller sequence ions, limiting the identification of complementary ions.^53,54^ As with intact proteins, the sequence coverage of intact gRNA and the detection of ions could be improved via the use of alternative fragmentation techniques, higher-resolution mass analyzers, or ion mobility.^57−60^

### Automated Identification and Quantitation of gRNA Base Substitution Spacer Impurities

Further capitalizing on the advantages emergent from the automated and confident assignment of gRNA fragment ions, a workflow was developed to identify gRNA impurities, specifically single-base substitutions, to aid the automation of spacer fidelity assessments. Importantly, base substitutions are common impurities for synthetic RNA molecules^61^ entailing sequence inaccuracies that could promote unintended off-target edits if present in the spacer region. Thus, the automated fragment search workflow was leveraged to facilitate the discovery of aberrant single-base substitutions. This discovery strategy was performed by generating a sequence list of all possible single-base substitution permutations for the spacer region of the parent sequence (corresponding to a list length of *n* × 3, where *n* is the number of nucleotides in the spacer sequence) and searching for all theoretical fragments for each permutation; each substituted sequence was subsequently scored based on the fragment intensity as well as fragment cosine score and weighted by the possible sequence coverage afforded by unique *c-*fragments not shared with the parent sequence (*i.e.*, all *c-*fragments between *c*_*n*_ and *c*_*m*_, where *n* is the base substitution site and *m* is the final position in the spacer sequence), as described in eq 2. Due to the overlap between isotopic distributions created by the ±0.98 Da difference produced by C > U and U > C mutations, these single-base substitutions were excluded from this discovery analysis in favor of a more robust approach that accounts for isotopic overlap, as demonstrated in the subsequent section.

**Figure 3:**
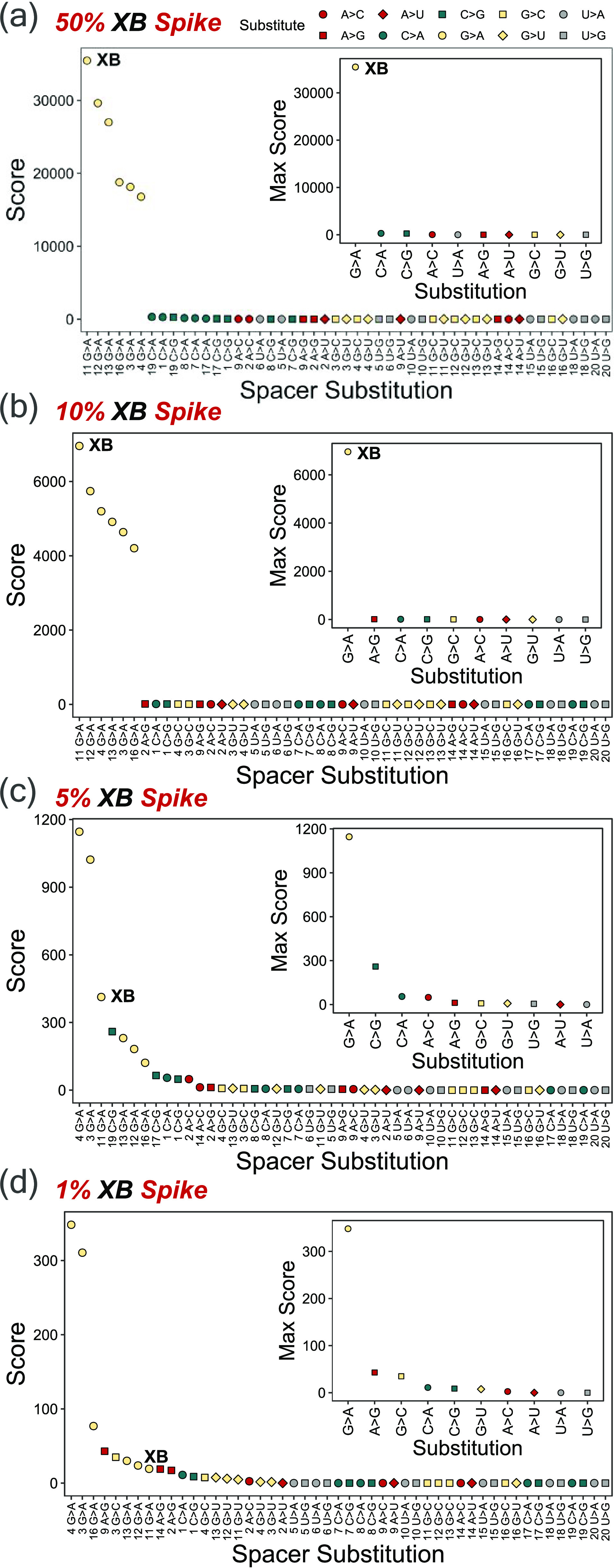
Top-down gRNA single-base substitution discovery: gRNA X was spiked with (a) 50%, (b) 10%, (c) 5%, and (d) 1% total concentrations of gRNA XB (11 G > A) and subjected to top-down MS/MS. Spectra were searched for all possible single-substitution variants, excluding C > U and U > C substitutions, and scored based on detected fragment ions. Labels on the *x-*axis indicate the substitution position followed by the type of substitution. Insets summarize results by displaying the max score for each substitution type, ignoring substitution position. Note differences in *y*-axis scales.

To assess the discovery of single-base substitutions via this approach, MS/MS spectra for mixtures of gRNA X spiked with variant XB at 1, 5, 10, and 50% total gRNA concentrations were each searched for all possible single-base substitution variants of gRNA X and scored individually. At a 50% gRNA concentration (Figure 3a), this approach readily identified variant XB (11 G > A) as the top-scored impurity. Additional G > A base substitutions at positions 12 and 13 also scored highly, followed by 16 G > A, 3 G > A, and 4 G > A; each of these assignments shares fragment masses with variant XB. For example, masses for *c*_11_–*c*_20_ for 3 G > A and 4 G > A are all isomeric to *c*_11_–*c*_20_ for the actual impurity, 11 G > A. By this effect, several fragment assignments and high scores were produced by the discovery approach for all G > A substitutions. However, this effect is mitigated when considering only the identity of the base substitution, as presented in the inset of Figure 3a, where only the max score for each base substitution is displayed, illuminating a clear presence of a G > A impurity that is identified at a vastly higher score (score of 3.5 E4) over the second highest impurity, C > A (score of 294). Regardless, at a 50% total gRNA concentration, variant XB was accurately identified as 11 G > A by the impurity discovery approach, correctly elucidating both the G > A base substitution and the substitution site. Similarly for the 10% spike-in experiment, the impurity could be confidently identified as variant XB (Figure 3b), again denoted by a high score for 11 G > A (score of 7.0 E3) over all other impurities, especially non-G > A substitutions, which presented a max score of 12 for A > G. Although the base substitution can no longer be localized at a 5% total gRNA XB concentration (Figure 3c), evidenced by the higher score for 4 G > A and 3 G > A over 11 G > A, the correct base substitution of G > A is still assigned as the highest-scoring impurity. Likewise, for the 1% gRNA XB spike-in (Figure 3d), the localization of the base substitution was challenged, yet the G > A substitution remained the highest-scoring impurity. Overall, gRNA X spiked with variant XB demonstrated that this method enables the discovery of single-substitution spacer impurities even at a 1% impurity concentration as well as the localization of the substitution site at a 10% impurity concentration, yielding a strategy that enables the rapid discovery of low-abundance impurities and enhances the available toolset for synthetic gRNA spacer characterization.

Further assessments of the impurity discovery strategy were performed by evaluating gRNA X spectra spiked with variants XA or XC, which featured base substitutions near the 5′-end (position 2) and 3′-end (position 20) of the spacer region, respectively. For variant XA spiked in at 50 and 10% total gRNA concentrations (Figure S8a,b), facile assignment and localization of the base substitution impurity as 2 A > U, aligning correctly with the sequence of variant XA, remained possible. At a 5% total gRNA concentration (Figure S8c), the A > U substitution was outscored by 19 C > A. Substitutions near the 3′-end of the spacer, such as at position 19 or 20, may be falsely identified due to the low number of unique *c*-fragment ions that would be produced by these sequences. For example, a spacer with a mutation at position 20 would only produce one unique *c-*fragment that would be critical to identify the substitution; however, if the unique fragment ion is detected due to a false assignment, then the substitution becomes identified at a high score by the discovery approach, as noted in Figure S8c, where 19 C > A was identified due to the false assignment of one *c*_20_ fragment. At a 1% total gRNA concentration (Figure S8d), 2 A > U could not be confidently identified due to a sparse coverage for variant XA. For variant XC, Figure S9a,b demonstrates that the impurity can be correctly assigned as 20 U > G when present at a 50 and 10% total gRNA concentration. However, variant XC was not detected in the 5 or 1% mixtures (Figure S9c,d), again resulting from low sensitivity for larger fragment ions and the need to detect the critical and only unique fragment ion, *c*_20_, to assign impurities with substitutions at position 20. Even though impurity discovery for base substitutions at the fringes of the spacer region was not as sensitive as for variant XB, correct base substitution assignment and localization were still possible at a 10% concentration, nevertheless facilitating the rapid discovery of impurities that may guide orthogonal characterization strategies. These results highlight the power of mass spectrometry to quickly assess spacer fidelity and motivate the development of more sensitive mass spectrometry workflows to surpass the merits unveiled in these exploratory analyses.

### Automated Quantitation of Low-Level C > U and U > C Substitutions by Isotopic Profile Matching

**Figure 4:**
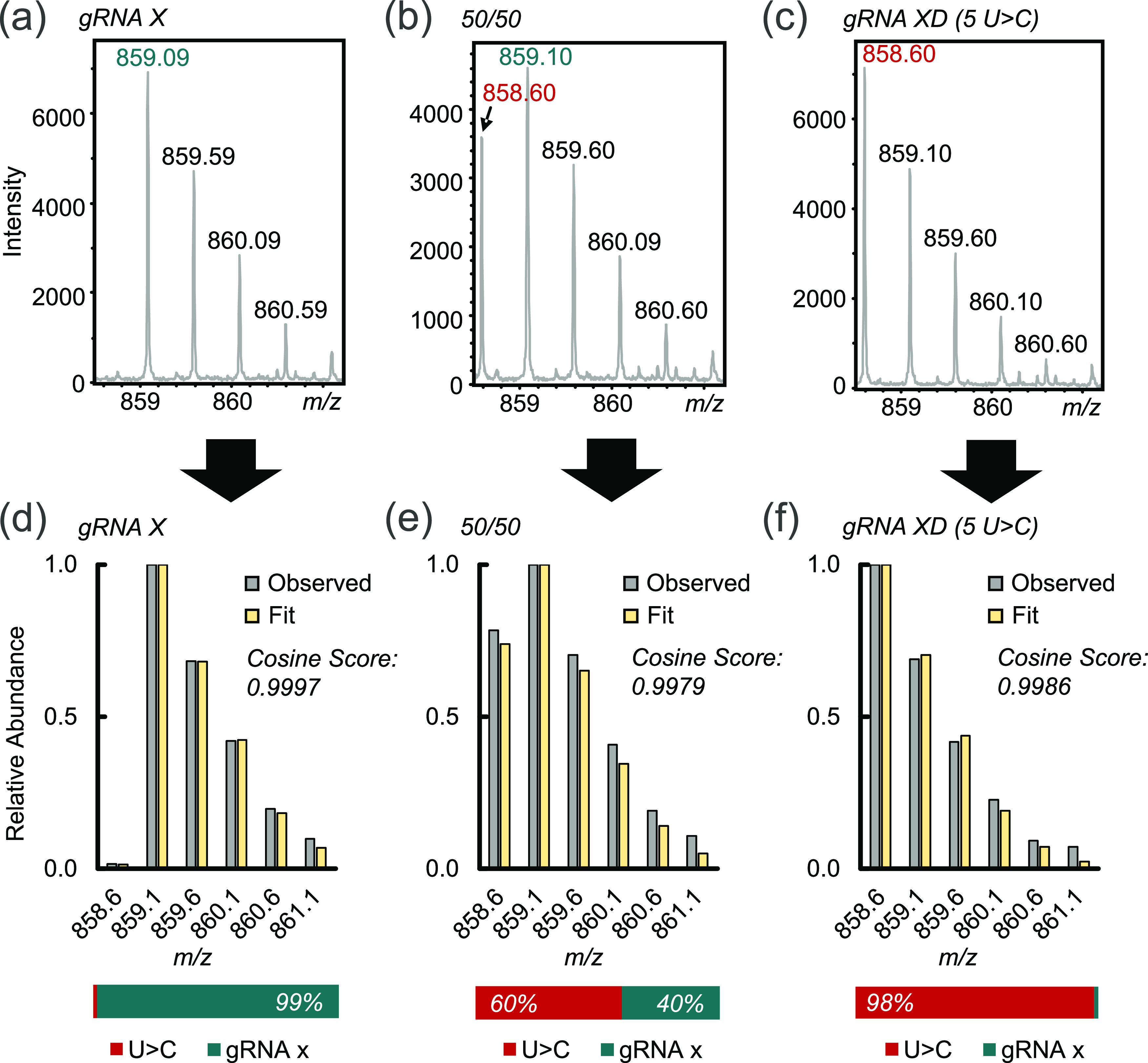
Top-down gRNA spacer sequence quantitation of U > C or C > U substitutions: zoom of isotopic profiles for fragment *c*_5_^2–^ of (a) 100% gRNA X, *(*b) a 50:50% mixture of gRNA X and a 5 U > C sequence substitute (gRNA XD), and (c) 100% gRNA XD. Each isotopic profile was interpreted as a mixture of both sequences to generate optimal fits for (d) gRNA X, (e) 50:50 mixture of gRNA X and gRNA XD, and (f) gRNA XD. The monoisotopic peaks for gRNA X and gRNA XD are shown in teal and red fonts, respectively.

Additional advancements to gRNA spacer characterization and quantification were achieved by devising a strategy to distinguish C > U and U > C substitutions that would otherwise confound sequence assignments. Although the monoisotopic masses of U nucleotides differ from C nucleotides by 0.984 Da, a mass difference readily resolved at the mass resolution afforded by TOF detectors, the mass difference between the carbon-13 isotope of C nucleotides differs by only −0.019 Da from the monoisotopic mass of U nucleotides, resulting in signal interference that ultimately distorts fragment ion isotopic distributions. Figure 4 demonstrates these changes in isotopic distribution of the *c*_5_^2–^ fragment for gRNA X, for variant XD featuring a U > C substitution at position 5, and for a 50/50 mixture of both gRNA sequences. While the *c*_5_ fragments for variant XD and gRNA X may be unambiguously assigned through the detection of the monoisotopic peaks in Figure 4a,c, respectively, the 50/50 gRNA mixture, however, presents a distorted isotopic distribution that hinders the assignment of the *c*_5_ fragment ion and disables the critical ability to quantify relative abundances in the mixture using fragment ion abundances. Addressing this shortcoming in quantifying C > U or U > C impurities necessitates a method capable of inferring fragment ion identities and abundances from overlapping isotopic distributions.

Accordingly, an automated data analysis strategy was implemented to model observed *c-*fragment ion distributions as a theoretical mixture of the parent gRNA sequence and either a U > C or C > U substitute. By generating theoretical isotopic distributions for the target and substituted sequence *in silico*, theoretical mixtures of both isotopic distributions can be constructed to solve for a fit that best matches the observed distribution using a combination of non-negative least-squares fitting followed by minimization of the cosine distance between the fit and observed isotope abundances. Percentages of each sequence (target and substitute) contributing to the best fit are accordingly indicative of the in-solution relative abundance of each sequence. Figure 4 demonstrates this analysis for the *c*_5_ fragment isotope distribution of gRNA X, variant XD, and the 50/50 mixture of both guides produced by fitting each distribution to a mixture of the gRNA X sequence and a U > C variant. Subjecting the observed *c*_5_^2–^ isotope distribution produced for gRNA X in the absence of the U > C substitute resulted in a theoretical best fit composed of 98.6% of the gRNA X theoretical isotope distribution and 1.4% of the U > C theoretical distribution (Figure 4a), approximating the actual composition of 100% gRNA X. Similarly for the *c*_5_^2–^ isotope distribution of the U > C substitute in the absence of gRNA X, the best-fit distribution was composed of 98.2% U > C and 1.8% of gRNA X (Figure 4c), again resembling the actual abundance of 100% U > C. Analysis of the 50/50 mixture of both gRNA sequences resulted in a theoretical fit composed of 59.9% U > C and 40.1% gRNA X (Figure 4b), not only providing identification of both sequences but also bestowing a reasonable estimation of relative in-solution concentration that would otherwise be impossible to measure.

To further cement the quantitative capabilities of this analysis, the U > C substitute was spiked into gRNA X at 1, 5, 10, and 50% total gRNA concentrations prior to top-down MS/MS of each mixture. The resulting spectra were searched for *c*_5_, *c*_10_, *c*_15_, and *c*_20_ isotope distributions that were each fitted as a mixture of the gRNA X and U > C substitute sequences. The percent abundance of the U > C mutant determined by MS/MS isotope fitting was plotted as a function of the in-solution percent gRNA concentration, as shown in Figure S10a. For all of these fragments, the U > C abundance estimated by the isotope fit correlated with the in-solution concentration with good accuracy across the spacer region, further testifying to the utility of this methodology to characterize and quantify spacer fidelity. To demonstrate that this approach is also applicable to evaluate C > U substitutions (instead of U > C), the inverse experiment, where gRNA X was spiked into variant XD at low concentrations, was performed. Plotting the abundance of gRNA X, representing a C > U substitute, as a function of its concentration also resulted in acceptable quantitation for each fragment evaluated across the spacer sequence (Figure S10b). Altogether, the capabilities of MS/MS to assess spacer fidelity are ultimately enhanced by utilizing information encoded into isotopic distributions to identify and quantify potential C > U and U > C sequence substitutions that would otherwise remain uncharacterized.

Expanding upon this approach, the isotopic evaluation of *c*-type fragments was implemented to identify C > U and U > C single-base substitutions for all detected fragment ions. In Figure S11, the same isotopic fitting method was applied to uncover U > C substitution impurities in gRNA X spiked with variant XD at 1, 5, 10, and 50% and evaluate the isotopic distribution of all *c-*type fragments across the entire spacer region. Analogously, Figure S12 displays results for the analysis of variant XD spiked with gRNA X to evaluate the discovery of C > U base substitutions. For the 50% spike-in mixtures, U > C and C > U substitutions are readily identified and localized to position 5, as displayed in Figures S11 and S12, respectively, based on the lack of substitution detection from *c*_1_ to *c*_4_ followed by a high abundance of the respective base substitution from *c*_5_ to *c*_20_. At the 10, 5, and 1% U > C spike-in, a similar trend is observed where a lack of substitution is observed at the first 4 nucleotides (Figure S11), contrasting with a mostly consistent detection of the substitution for the remainder of the spacer region. Averaging the percent U > C detected across positions 5–20 for each mixture results in mean U > C percentages that decrease as the in-solution concentration of variant XD decreases (Table S9), again demonstrating the adequate relative quantitation of these base substitutions. Likewise, for C > U substitution searches, a mild abundance of base substitution was detected for *c*_5_–*c*_20_ in the 10, 5, and 1% spike-in of gRNA X into variant XD (Figure S12), with average abundances across these fragments tabulated in Table S9 to show decreasing abundances of C > U as the concentration of gRNA X decreased. It is noted that even when no U > C or C > U substitutions were present in solution (0% spike-in from Figures S11 and S12), low abundances of U > C and C > U were still detected, particularly for larger *c*-fragments; these misassignments likely arise from lower sensitivity for these fragment ions, resulting in isotopic distributions that may be easily distorted by noise or interference. Integration of more advanced deconvolution strategies, nano-LC, or gas-phase separation techniques, such as ion mobility, could serve to address false assignments, improve sensitivity, and ultimately augment the current capabilities of the novel U > C/C > U substitution discovery strategy developed here to provide an unprecedented high-throughput characterization of gRNA spacers with single-nucleotide resolution.

### *De Novo* Sequencing of gRNA Spacer Sequences and Low-Abundance Impurities with Highly Dissimilar Sequences

**Figure 5:**
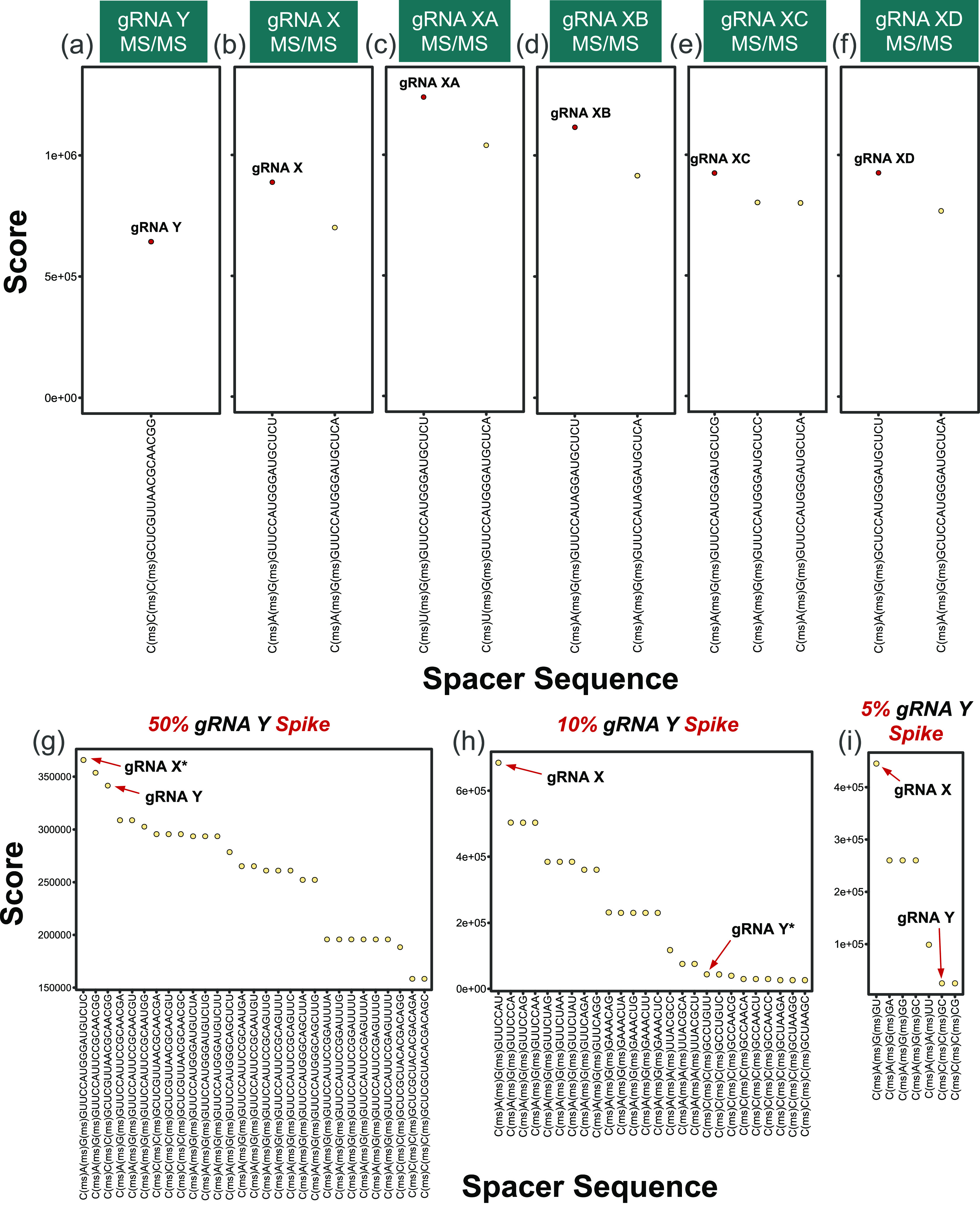
Top-down gRNA spacer *de novo* sequencing: scores for all identified sequences identified through *de novo* analysis of top-down MS/MS spectra of (a) gRNA Y, (b) gRNA X, (c) gRNA XA, (d) gRNA XB, (e) gRNA XC, and (f) gRNA XD. Identified sequences that match the gRNA spacer are denoted by red points. (g) Scores for all identified sequences identified through *de novo* analysis of the first 20 nucleotides for top-down MS/MS spectra of gRNA X spiked with 50% gRNA Y. (h) Scores for all identified sequences identified through *de novo* analysis of the first 10 nucleotides for top-down MS/MS spectra of gRNA X spiked with 10% gRNA Y. (i) Scores for all identified sequences identified through *de novo* analysis of the first 5 nucleotides for top-down MS/MS spectra of gRNA X spiked with 5% gRNA Y. N(ms) denotes a phosphorothioate backbone at the 3′-end of the preceding 2′-O-methylribonucleotide. * indicates that a sequence tag was identified with minor inaccuracies. Note differences in *y*-axis scales.

Exploiting the thorough and robust gRNA spacer characterization afforded by these top-down MS/MS analyses, the automated fragment searches, base substitution discovery, and C > U/U > C distinction strategies were combined to further elevate the top-down methodology to perform *de novo* sequencing of gRNA spacer sequences and rapidly assess gRNA samples even when sequences are unknown. Deploying this strategy involves constructing a theoretical spacer sequence *in silico* composed of “empty” nucleotides (informing only the position of methylation and phosphorothioate modifications) that serve as placeholders that are substituted out, in the 5′-to-3′ direction, for A, G, C, or U nucleotides; if a matching *c-*fragment is detected for the substituted sequence, the respective base substitution is recorded as a new sequence and assigned a score that is updated as the search progresses. In cases where either a C or U nucleotide is identified, the isotopic profile of the fragment is evaluated to confirm the assignment, negate the assignment, or assign the identification as a mixture of sequences differing by a C > U/U > C substitution. Essentially, the strategy searches for all possible *c*_1_ fragments and generates a sequence list based on those identifications that is iterated upon to search for and identify all possible *c*_2_ fragments to again generate a new sequence list for which the process is repeated, until the end of the spacer (*c*_20_) is identified, resulting in the identification of all spacer sequences with a 100% sequence coverage. After the spacer sequence is constructed out to 5, 10, 15, and 20 nucleotides, the identification list is scored by eq 4 and reduced to only the best-scoring sequence for every set of isomeric identifications, aiming to mitigate the presence of sequence scrambles. Applied to top-down MS/MS spectra of intact gRNA, this *de novo* sequencing strategy was able to accurately identify the spacer sequences for all of the gRNA samples evaluated. Analysis of top-down MS/MS spectra of gRNA Y resulted in the identification of one spacer sequence that mapped perfectly to the gRNA sequence (Figure 5a), while MS/MS spectra for gRNA X and its variants (Figure 5b–f) each resulted in the identification of 2–3 spacer sequences corresponding to the actual sequence and base substitutions at position 20, which are caused by misassignments of *c*_20_ fragments, as discussed for the single-base substitution results. Regardless, the exact spacer sequence was correctly identified as the highest-scoring sequence in each of these analyses, establishing *de novo* MS/MS sequencing as a promising method for evaluating unknown or unexpectedly contaminated gRNA spacer sequences.

Ultimately, such a *de novo* approach would assist with the discovery of sequence impurities featuring not just single-base substitutions but rather highly dissimilar spacer sequences with multiple base substitutions throughout the spacer. Providing a proof-of-concept, MS/MS spectra for gRNA X and gRNA Y mixed at multiple concentration ratios were subjected to a *de novo* search modified to identify lower-abundance impurities that may fail to generate detectable fragment ions in the mixed spectra and stifle sequence coverage. To accommodate the detection of low-abundance impurities via a *de novo* search, the modified strategy allowed for 1 missed fragment for every 5 nucleotides to relax the 100% sequence coverage requirement but still demands the identification of *c*_1_, *c*_*2*_, and *c*_*3*_ fragment ions, which are typically the most abundant in the top-down MS/MS spectra (shown in Figure 1d for gRNA X). The resulting scores for all sequence assignments were then weighed by the final sequence coverage. *De novo* sequencing for the 50% mixture yielded 29 spacer sequences (Figure 5g); the top-scoring sequence corresponded to the gRNA X spacer, except that nucleotides 17–20 were assigned as “UCUC” instead of “CUCU”. Inspection of the spectra revealed that this misassignment is due to low abundance and distorted isotopic profiles for *c*_17_ and *c*_19_, impeding the automated detection and distinction of C/U nucleotides. In contrast, the full sequence for gRNA Y was accurately detected as the third highest-scored sequence assignment, without presenting any such sequence scrambles. The second highest-scored sequence matched the gRNA X sequence from nucleotides 1 to 10 and gRNA Y from nucleotides 13 to 20, resulting in a high score for a nonexistent sequence. Although this and several additional nonexistent sequences were identified, such an observation is expected to occur when allowing for sequence identifications with a <100% sequence coverage.

On evaluating a mixture composed of 90% gRNA X and 10% gRNA Y to assess sensitivity, it was not possible to map out the low-abundance gRNA Y spacer for all 20 nucleotides due to poor detection of larger *c*-fragments (*c*_15_–*c*_18_ were not detected) and concomitant low sequence coverage for the lower abundance gRNA. However, mapping out only half of the spacer region, nucleotides 1–10, allowed for the detection of sequence tags for both gRNA spacers, as shown in Figure 5h. In total, 26 distinct sequences were identified, where the highest-scoring sequence mapped perfectly to nucleotides 1–10 of gRNA X followed by several sequences that share *c-*ion fragment masses with gRNA X. Furthermore, nucleotides 1–10 for gRNA Y were identified even at a 10% total gRNA concentration with only one sequence inaccuracy, where nucleotides 6 and 7 were assigned as “CU” instead of “UC”. Detecting gRNA Y at a 5% total gRNA concentration was achieved by further reducing the length of the sequence search to only nucleotides 1–5. Regardless, identifying this shorter sequence tag for gRNA Y by *de novo* sequencing was nonetheless possible, even at a 5% concentration (Figure 5i). Interestingly, plotting sequence scores (prior to weighing by sequence coverage, as described in eq 3) for each gRNA Y sequence tag as a function of the gRNA Y concentration in each mixture reveals a linear relationship (Figure S13), highlighting the quantitative merits of the top-down strategy that are retained even in a *de novo* approach.

Altogether, these results favor an overall positive outlook on the use of *de novo* sequencing of gRNA impurities by top-down mass spectrometry. Even though issues including the incomplete coverage of spacer, misassignment of C/U nucleotides, and assignment of nonexistent sequences currently persist, these limitations originate from inadequate production and detection of sequence fragments, particularly larger RNA *c-*fragments that would inform deeper into the spacer region. Foreseeably, devising MS/MS strategies tuned to achieve greater sensitivity for larger RNA fragment ions would serve to address the majority of the challenges encountered. In recent years, advanced strategies integrating chemical derivatization, nano-ESI, ion/ion reactions, ion mobility, and alternative ion activation strategies have eliminated similar challenges in top-down proteomics,^57−60^ pushing the field as a premier method in state-of-the-art biophysical characterization; fortifying oligonucleotide top-down mass spectrometry by adopting these advanced strategies promises to surpass current limitations of *de novo* sequencing and elucidate unexpected, yet critical, impurities to meet the increasing demands for exceptional gRNA spacer fidelity from the enlarging field of CRISPR-based therapeutics.

### *De Novo* RNA Modification Assignment and Localization

Further capitalizing on the wealth of information present within the rich MS/MS spectra, RNA modification localization by top-down MS/MS was also amended into the RNA characterization strategy, as this methodology is positioned to provide unique insights into modification discovery for large 100-mer sequences. As discussed above and illustrated in Figures S6 and S7, top-down MS/MS spectra provided good coverage of the gRNA sequences, even for the highly modified tracr region, promising adequate localization capabilities of the 2′-O-methylation and phosphorothioate modifications present within these sequences. Importantly, in order to pinpoint an RNA modification to a specific nucleotide position, the nucleotide must preferably be flanked by adjacent fragment ions. For example, fragments *y*_2_ and *y*_3_ flank nucleotide U98 and allow the 2′-O-methylation and phosphorothioate modification to be localized to this site. Consequently, in order to unequivocally localize all RNA modifications with single-nucleotide resolution, an ideal 100% sequence coverage would be necessary. In instances where a modified nucleotide position is not immediately flanked by fragment ions, such as 2′-O-methylated G21 where *c*_20_ is detected while *c*_21_ is not (Figures S6 and S7), the modification can become more ambiguous if the modification location is not already known; in the case for gRNA X (Figure S6a), because *c*_22_ was also undetected while *c*_23_ was identified, the 2′-O-methylation can only be assigned to occur within the sequence stretch spanning position 21 to position 23. For all other gRNA (Figure S7), *c*_22_ was detected, allowing the 2′-O-methylation modification to be assigned to a slightly shorter sequence stretch spanning positions 21 and 22. Although in such cases single-nucleotide resolution is not achieved, the identification of a modification at a specific sequence stretch remains valuable, as the presence of gRNA modifications can be otherwise difficult to uncover by orthogonal strategies such as NGS.

To exemplify these concepts, RNA modification characterization via MS/MS was demonstrated by the development of a *de novo* RNA modification assignment method that applied these top-down modification localization strategies. In this approach, both the MS/MS spectra and the nucleotide sequence with no modification information were provided to a modified version of the *de novo* sequencing tool described above that was instead programmed to search for and assign 2′-O-methylation and phosphorothioate modification sites up to position 28, via identification of *c-*type fragments. Coverage by *c-*ions beyond position 28 was sparse, negatively impacting localization, as described above. For this approach, complete coverage from positions 1 to 20 was required and only 1–2 missed cleavages from positions 21 to 28 were allowed to consider a *de novo* hit. Sequence base substitutions were not considered in this iteration to instead solely focus on RNA modification assignments. Although only *c-*ions were examined, an analogous analysis of *y-*type fragments could foreseeably be applied to localize modifications on the 3′-end. The resulting *de novo* RNA modification assignments are shown in Figure S14. For gRNA XA, the highest-scoring sequence correctly identified and localized all modifications on the first 28 nucleotides on the 5′-end, which includes 2′ O-methylation at positions 1, 2, 3, 21, and 28 as well as phosphorothioate modifications between positions 1–2, 2–3, and 3–4. As discussed, the lack of detection for fragment *c_21_* led to the assignment of 2′ O-methylation at position 22 instead of position 21 in some of the other gRNA XA *de novo* assignments with slightly lower scores. Misassignment of this modification was also observed for gRNA XC, gRNA XD, and gRNA Y where the only *de novo* hit correctly assigned all modifications and their locations with the exception of 2′ O-methylation at position 22 instead of at position 21, again due to a lack of complete sequence coverage. Nonetheless, the presence of the modification can still be confidently identified, even if single-nucleotide resolution is not achieved. For gRNA X, fragment *c*_22_ was undetected; thus, the methylation modification at position 21 was instead assigned at position 23, while for gRNA XB, fragment *c_28_* was not detected, so the methylation modification at that position was also not identified or localized. Despite some incomplete sequence coverage, which may be resolved in future experiments, the assignment of RNA modification by top-down MS/MS is nevertheless informative. As with top-down proteomics, the main hindrance to unambiguous assignments of nucleotide or modifications stems from sequence coverage below the ideal 100%. Even for the more mature field of top-down proteomics, achieving complete sequence coverage and unambiguous localization of modifications remain unresolved problems but have slowly been ameliorated with the development of novel approaches that would likely also serve to improve RNA characterization.^57−60^ A secondary issue also arises from modifications with identical masses. As an example, 2′ O-methylation of an RNA nucleotide would be indistinguishable from methylation of the nucleotide bases in N6-methyladenosine (m^6^A) or 5-methylcytosine (5-mC). Isomeric amino acids, such as leucine/isoleucine and aspartic acid/iso aspartic acid, pose similar challenges to top-down proteomics but have been addressed by utilizing MS^n^ and electron-based ion activation strategies^62,63^—two methodologies that could be similarly applied to mitigate potential issues with RNA modification identification.

## Conclusions

Remarkable advances in CRIPSR-based gene editing continue to illuminate new frontiers in biotechnology for disease treatment. Transforming these novel gene editing technologies into therapeutics necessitates the robust and detailed characterization of the drug product. Importantly, constituent gRNA spacer sequences in these biotherapeutics will specify the editing site for which highly detailed assessments of spacer fidelity are desired to ensure on-target edits and mitigate undesirable off-target effects. Aiming to elevate contemporary methodologies for gRNA spacer characterization, top-down mass spectrometry was herein implemented, automated, and evaluated to provide rapid, robust, sensitive, and quantitative characterization of gRNA spacer sequences. Extending beyond simple sequence confirmation, top-down MS/MS methodology demonstrated quantitative merits not achievable by orthogonal gRNA characterization methods, such as NGS, by expressing adequate relative concentration estimations of different sequences via comparison of fragment ion intensities, even at low concentrations. Furthermore, automated spectral interpretation empowered the high-throughput discovery of single-base substitution impurities through a database search as well as rapid identification of highly dissimilar spacers via *de novo* sequencing. The success of these strategies is, in part, attributed to the careful interpretation of isotopic envelopes to properly assign C and U nucleotides that would otherwise introduce challenges to mass spectrometry analyses arising from similar masses and overlapping isotopic interferences. Although current limitations persist in these strategies that are especially exacerbated when characterizing gRNA present at concentrations near the sensitivity limits of the mass spectrometer, the majority of hindrances encountered originate from insufficient formation or detection of fragment ions; notably, related issues have also plagued the field of top-down proteomics, but those barriers have increasingly been overcome to elevate top-down proteomics as a premier and leading method for protein characterization. Advanced tools such as nano-LC, gas-phase ion mobility separations, spectral deconvolution, ion/ion reactions, and alternative ion activation techniques have all been implemented to improve the sensitivity of top-down proteomics and may also enhance top-down oligonucleotide characterization. The current incarnation of top-down gRNA spacer characterization presented herein lays the foundation for the use of MS/MS characterization for large oligonucleotides. As gene editing technology and additional oligonucleotide-based therapies continually prove their utility as effective biotherapeutics, the need for thorough, high-throughput, and quantitative characterization of large RNA becomes increasingly important. Top-down mass spectrometry presents an overall auspicious strategy to meet the demands presented by the flourishing field of CRISPR-based therapeutics for thorough assessments of gRNA spacer fidelity.

## Supporting Information Available

Zoom of gRNA XA *c*_2_ ion at a 0 and 1% concentration (Figure S1); NGS results (Figure S2); concentration of library yields for each gRNA (Table S1); fragment assignment statistics (Figure S3); zoom of overlapping isotopic envelopes (Figure S4); spacer region coverage maps (Figure S5); complete coverage map and annotated spectrum for gRNA X (Figure S6); complete sequence coverage maps for gRNA XA, gRNA XB, gRNA XC, gRNA XD, and gRNA Y (Figure S7); single-substitution discovery analysis performed for gRNA X spiked with gRNA XA (Figure S8); single-substitution discovery analysis performed for gRNA X spiked with gRNA XC (Figure S9); quantitation for U > C base substitution spiked into gRNA X and C > U base substitution spiked into gRNA XD (Figure S10); U > C base substitution detection across the spacer region (Figure S11); C > U base substitution detection across the spacer region (Figure S12); mean percent of U > C/C > U substitutes across fragments *c*_5_–*c*_20_ (Table S9); percentage scores for gRNA Y sequence tags as a function of the gRNA Y concentration (Figure S13); and scores for top-down *de novo* RNA modification assignments (Figure S14) (PDF)
List of all fragment ions identified in top-down MS/MS spectra using offset mass lists (Table S2); list of gRNA X fragment ions identified by top-down MS/MS (Table S3); list of gRNA XA fragment ions identified by top-down MS/MS (Table S4); list of gRNA XB fragment ions identified by top-down MS/MS (Table S5); list of gRNA XC fragment ions identified by top-down MS/MS (Table S6); list of gRNA XD fragment ions identified by top-down MS/MS (Table S7); and list of gRNA Y fragment ions identified by top-down MS/MS (Table S8) (XLSX)
