# Supplementary material for: Spacer Fidelity Assessments of Guide RNA by Top-Down Mass Spectrometry
Source: ACS Cent Sci. 2023 Jul 11;9(7):1437–52. doi: 10.1021/acscentsci.3c00289 (PMC10375574; doi:10.1021/acscentsci.3c00289)

Spacer Fidelity Assessments of Guide RNA by Top-Down Mass Spectrometry

Luis A. Macias,[1] Sara P. Garcia,[1] Kayla M. Back,[1] Yue Wu,[1] G. Hall Johnson,[2] Sekar Kathiresan,[1] Andrew M. Bellinger,[1] Ellen Rohde,[1] Michael A. Freitas,[2,3] James A. Madsen[1,*]

[1] Verve Therapeutics, 201 Brookline Avenue, Suite 601, Boston, MA 02215
[2] MassMatrix, Inc, 302 Corry Street, Yellow Springs, OH 45387
[3] The Ohio State University, 281 West Lane Avenue, Columbus, OH 43210
[*] Corresponding author email: jmadsen@vervetx.com

Supporting Information
Table of Contents

	Content	Page
**Figure S1**	Zoom of gRNA XA *c*2 ion at a 0% and 1% concentration	S2
**Figure S2**	NGS Results	S3
**Table S1**	Concentration of library yields for each gRNA	S4
**Figure S3**	Fragment assignment statistics	S5
**Figure S4**	Zoom of overlapping isotopic envelopes	S6
**Figure S5**	Spacer region coverage maps	S7
**Figure S6**	Complete coverage map and annotated spectrum for gRNA X	S8
**Figure S7**	Complete sequence coverage maps for gRNA XA, gRNA XB, gRNA XC, gRNA XD, and gRNA Y	S9
**Figure S8**	Single substitution discovery analysis performed for gRNA X spiked with gRNA XA	S10-S11
**Figure S9**	Single substitution discovery analysis performed for gRNA X spiked with gRNA XC	S12-S13
**Figure S10**	Quantitation for U>C base substitution spiked into gRNA X and C>U base substitution spiked into gRNA XD	S14
**Figure S11**	U>C base substitution detection across spacer region	S15-S16
**Figure S12**	C>U base substitution detection across spacer region	S17-S18
**Table S9**	Mean percent of U>C/C>U substitutes across fragments c5 to c20	S19
**Figure S13**	Percentage scores for gRNA Y sequence tags as a function of gRNA Y concentration	S20
**Figure S14**	Scores for top-down *de novo* RNA modifications assignments	S21

Supplemental Figures and Tables:

Figure S1. Zoom of gRNA XA c_2 ion in the MS/MS spectrum of gRNA XA spiked at a (a) 0%, and (b) 1% concentration into gRNA X. The monoisotopic peak for the gRNA XA c_2 fragment ion (theoretical *m/z* 670.0444) was not detected in the 0% gRNA XA spectrum.

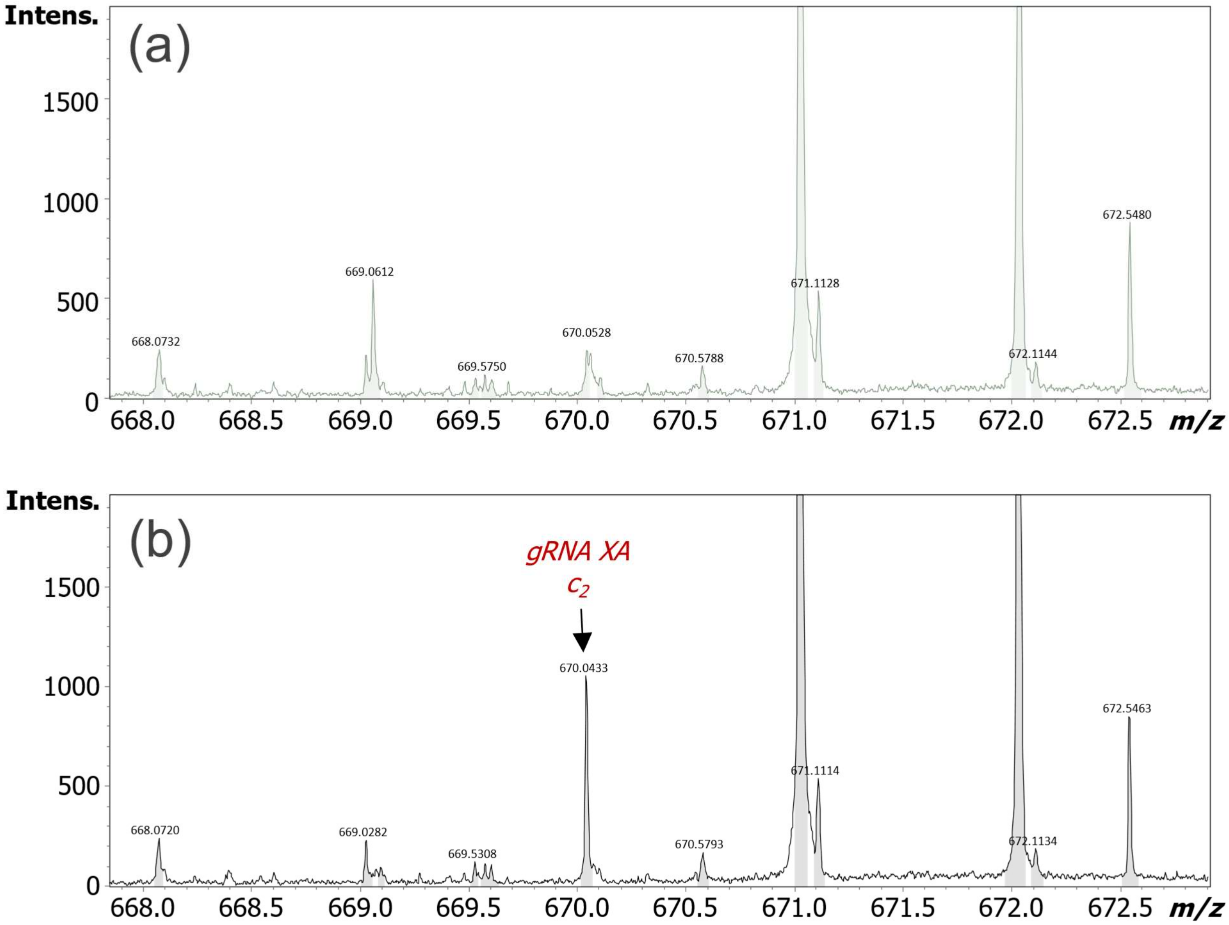

Figure S2. NGS analysis of gRNA: (a) The top read found by CRISPResso analysis of each gRNA library perfectly matches the respective gRNA nucleotide sequence. Number of matched reads are shown in parentheses. Sequences for the gRNA have been transcribed into DNA to serve as the reference. (b) TapeStation analysis of libraries prepared for NGS of each gRNA by reverse transcription – polymerase chain reaction. Expected size is 253 bp accounting for the addition of Illumina adapters and indexes (153 bp) to 100-mer gRNA. The no-template-control is labelled as NTC. Average library yields are listed in **Table S1**.

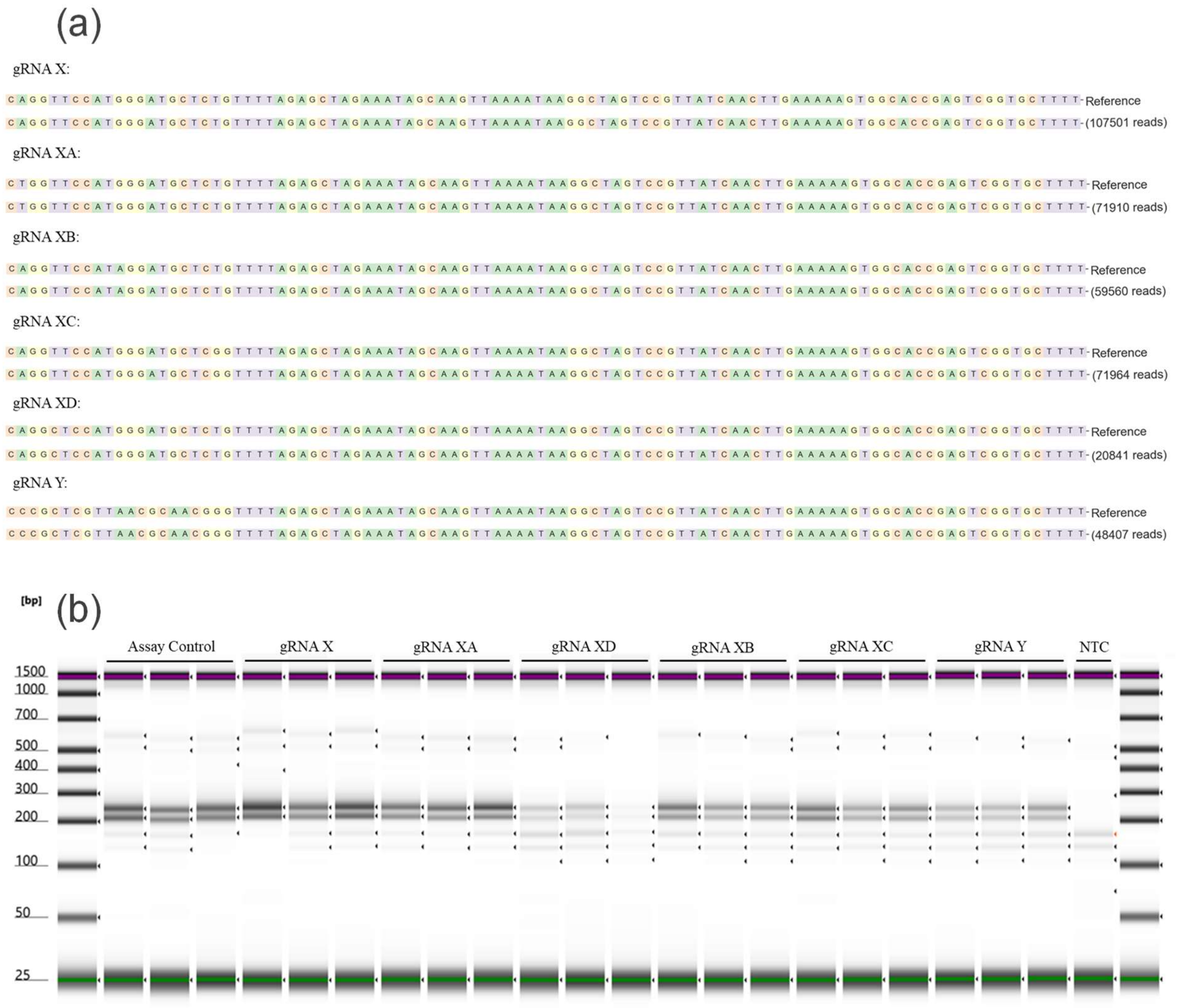

Table S1. Concentration of library yields for each gRNA.

gRNA	Run	Concentration (ng/µL)	Average Concentration (ng/µL)
gRNA X	1	5.93	5.26
gRNA X	2	4.29	
gRNA X	3	5.57	
gRNA XA	1	4.38	4.64
gRNA XA	2	4.37	
gRNA XA	3	5.17	
gRNA XB	1	4.18	3.35
gRNA XB	2	2.84	
gRNA XB	3	3.02	
gRNA XC	1	4.42	3.38
gRNA XC	2	2.60	
gRNA XC	3	3.13	
gRNA XD	1	1.72	1.34
gRNA XD	2	1.81	
gRNA XD	3	0.48	
gRNA Y	1	2.07	2.23
gRNA Y	2	2.09	
gRNA Y	3	2.53	

Figure S3. (a) Estimated false discovery rate for gRNA sequence fragments in mass shifted fragment lists before applying cosine score cutoff threshold (grey bars) and after applying a score threshold of 0.80 (teal bars). Horizontal dashed grey and teal lines indicate mean false discovery rate without and with a cosine score threshold, respectively. (b) Histogram of cosine scores for mass shifted random fragment assignments (purple bars) and non-mass shifted target hits (red bars). Vertical dashed black line denotes cosine score of 0.80.

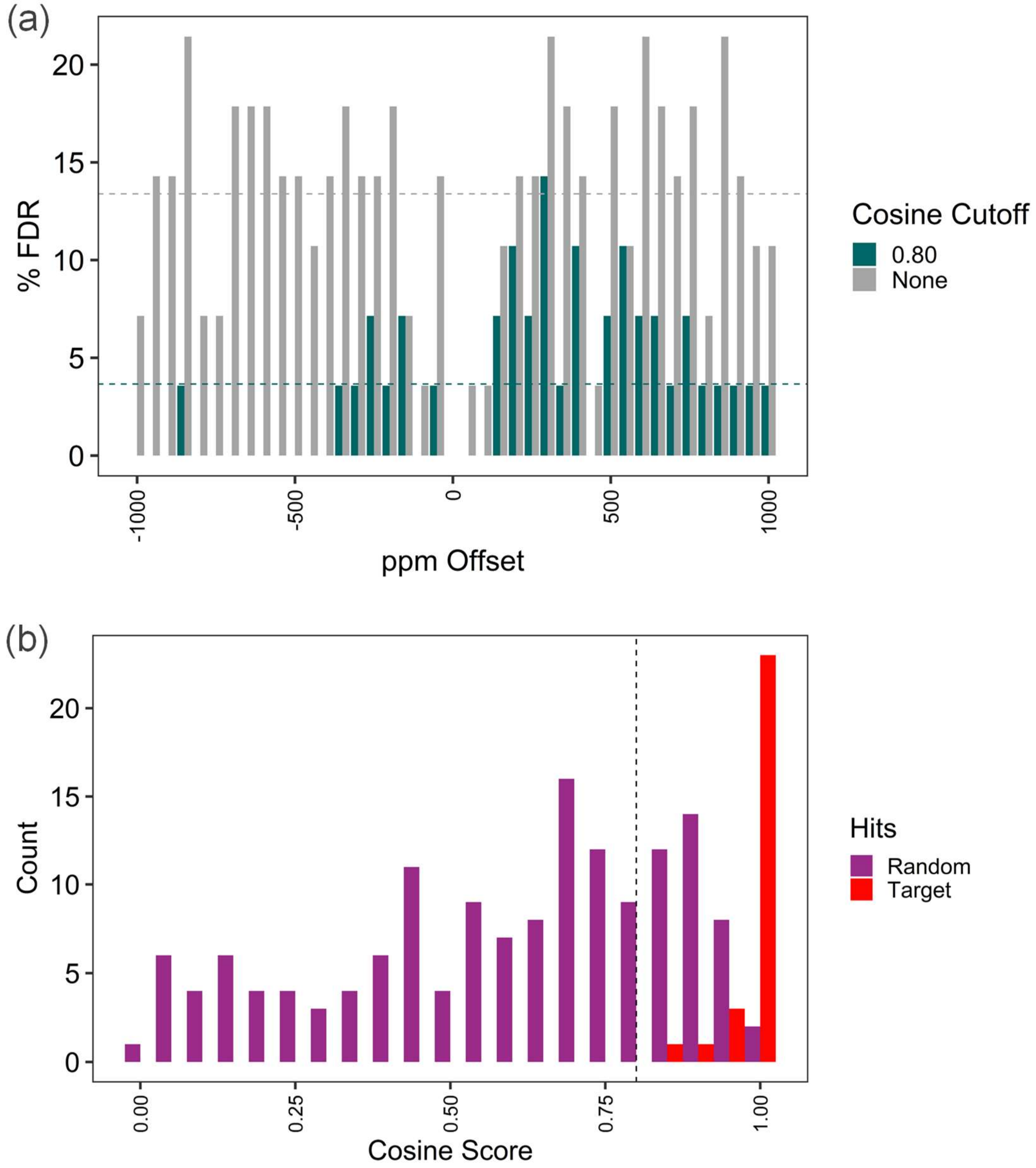

Figure S4. Zoom of isotopic envelope overlap of c_{15}^{5-} and c_9^{3-} in a top-down MS/MS spectrum of gRNA X.

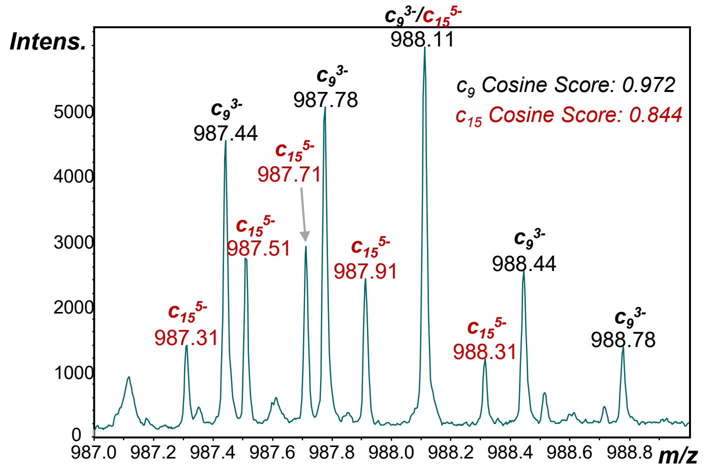

Figure S5. Automated top-down gRNA spacer sequencing identifies sufficient *c*-ion fragments to achieve full sequence coverage of all gRNA spacer regions evaluated. Fragment lists provided in **Tables S3-S8**.

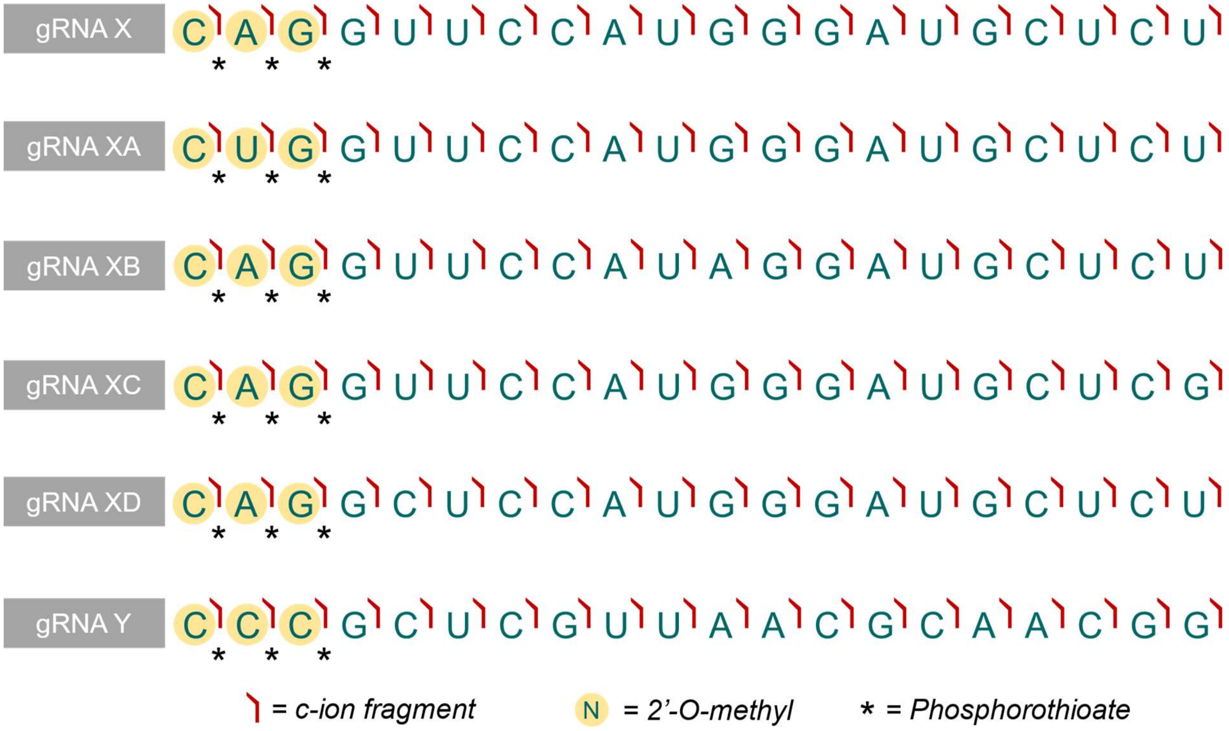

Figure S6. Complete sequence coverage map for (a) gRNA X and (b) annotated MS/MS spectra, zoomed on *m/z* 300 – 1310. Identified fragment ions are listed in **Table S3**.

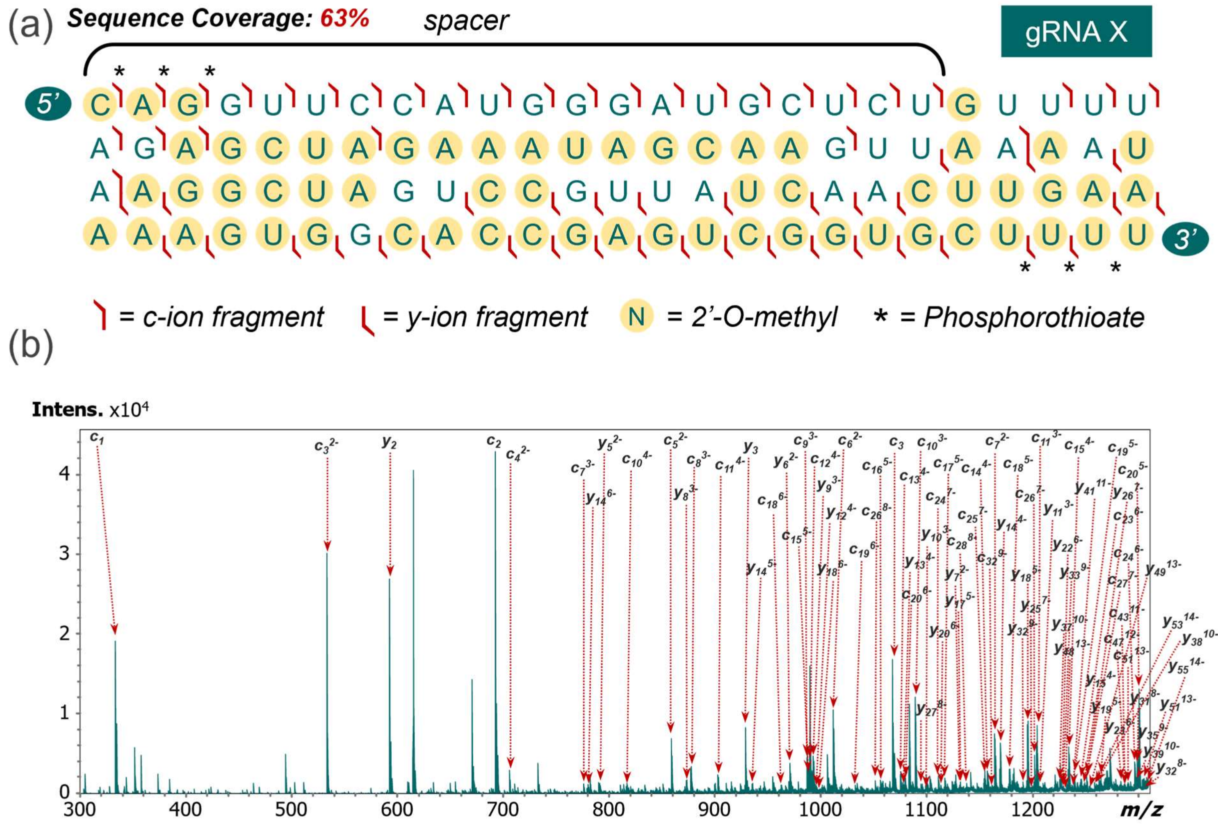

Figure S7. Complete sequence coverage maps for (a) gRNA XA, (b) gRNA XB, (c) gRNA XC, (d) gRNA XD, and (e) gRNA Y produced by top-down MS/MS. Fragment lists are provided in **Tables S4 – S8**.

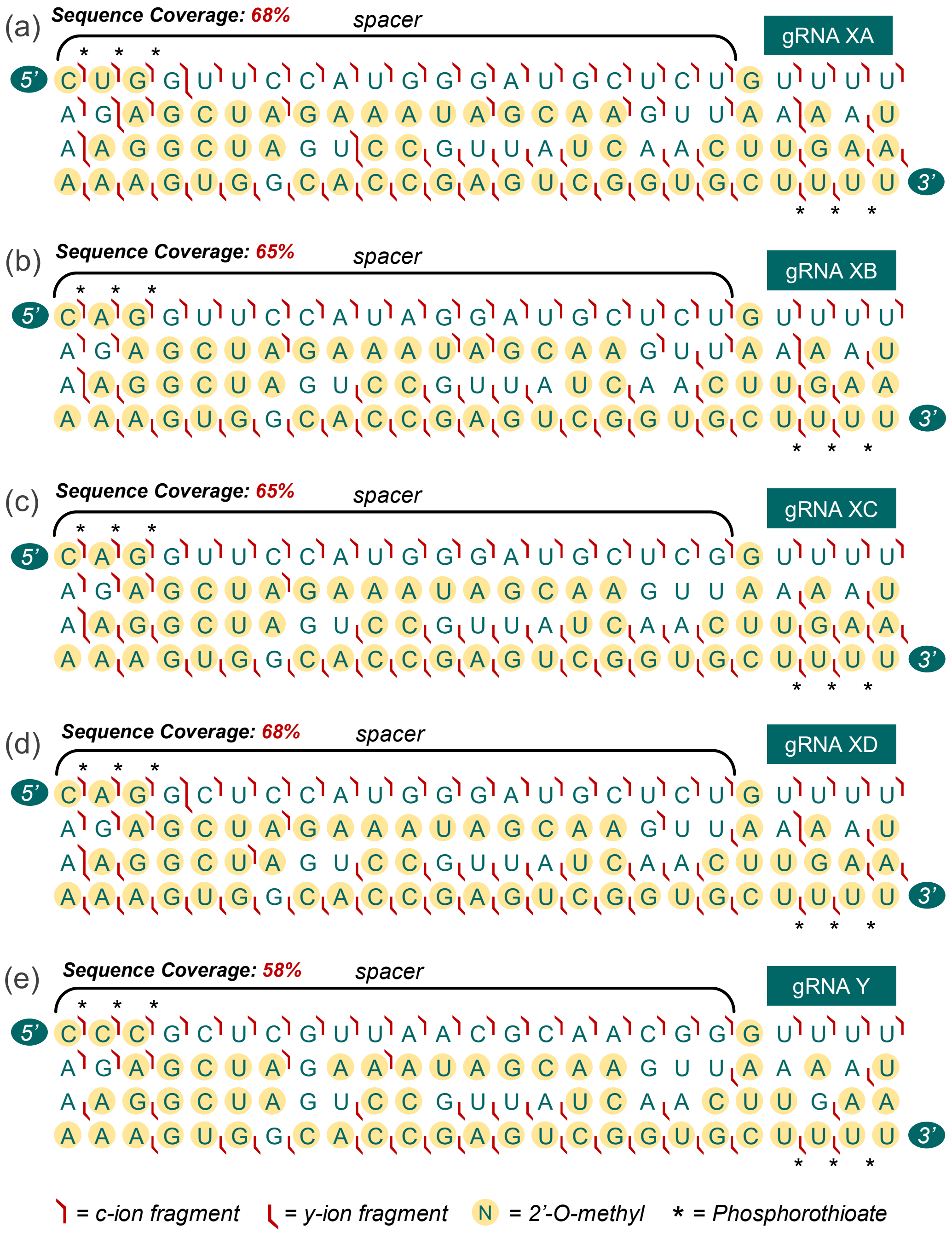

Figure S8. Top-down gRNA single-base substitution discovery: gRNA X was spiked with (a) 50%, (b) 10%, (c) 5%, and (d) 1% total concentration of gRNA XA (2 A>U) and subjected to top-down MS/MS. Spectra were searched for all possible single substitution variants, excluding C>U and U>C substitutions, and scored based on detected fragment ions. Labels on *x*-axis indicate the substitution position followed by the type of substitution. Insets summarize results by displaying the max score for each substitution type, ignoring substitution position. Note differences in *y*-axis scales.

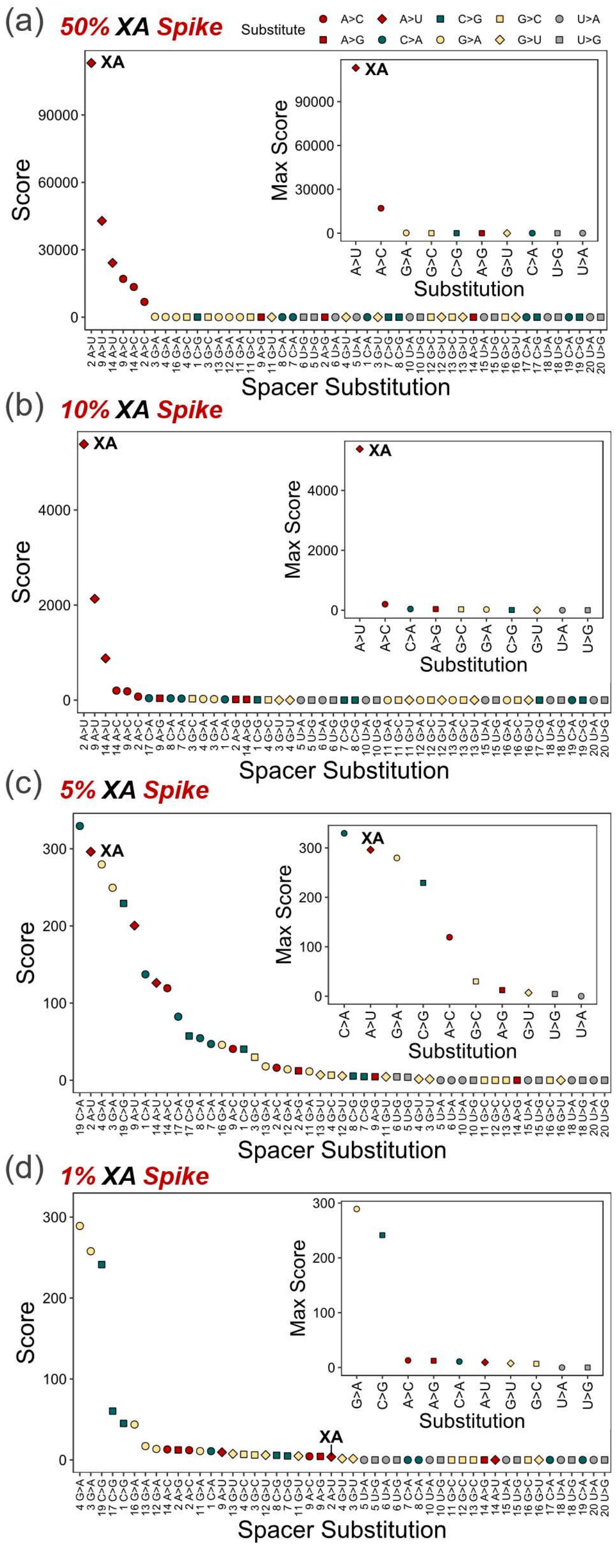
(a)
50% XA Spike
Substitute
A>C
A>U
C>G
G>C
U>A
A>G
C>A
G>A
G>U
U>G
XA
Score
Max Score
Substitution
Spacer Substitution
(b)
10% XA Spike
XA
Score
Max Score
Substitution
Spacer Substitution
(c)
5% XA Spike
XA
Score
Max Score
Substitution
Spacer Substitution
(d)
1% XA Spike
XA
Score
Max Score
Substitution
Spacer Substitution

Figure S9. Top-down gRNA single-base substitution discovery: gRNA X was spiked with (a) 50%, (b) 10%, (c) 5%, and (d) 1% total concentration of gRNA XC (20 U>G) and subjected to top-down MS/MS. Spectra were searched for all possible single substitution variants, excluding C>U and U>C substitutions, and scored based on detected fragment ions. Labels on *x*-axis indicate the substitution position followed by the type of substitution. Insets summarize results by displaying the max score for each substitution type, ignoring substitution position. Note differences in *y*-axis scales.

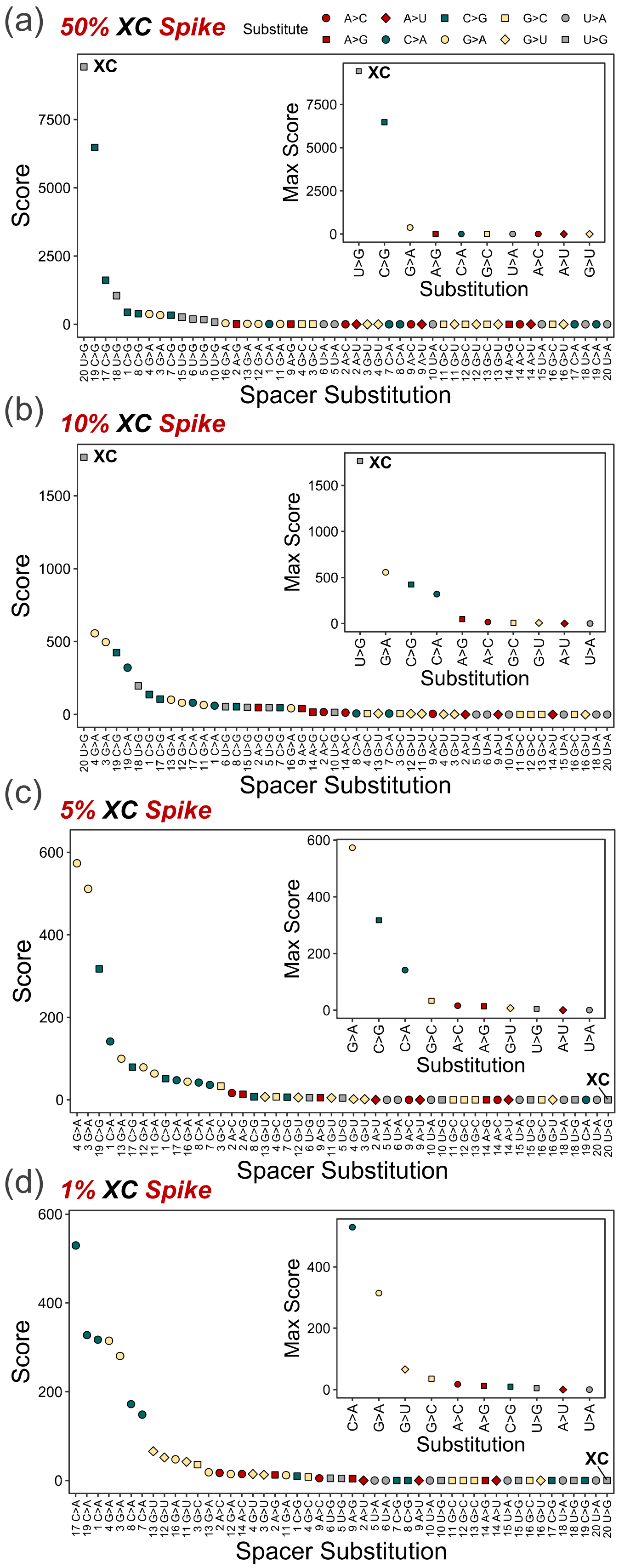
(a)
50% XC Spike
Substitute
A>C
A>G
A>U
C>A
C>G
G>A
G>C
G>U
U>A
U>G
XC
Score
Max Score
Substitution
Spacer Substitution
(b)
10% XC Spike
XC
Score
Max Score
Substitution
Spacer Substitution
(c)
5% XC Spike
XC
Score
Max Score
Substitution
Spacer Substitution
(d)
1% XC Spike
XC
Score
Max Score
Substitution
Spacer Substitution

Figure S10. Top-down gRNA spacer sequencing quantitation for (a) U>C base substitution spiked into gRNA X at and (b) C>U base substitution spiked into gRNA XD at 1%, 5%, 10%, and 50% total gRNA concentration, as determined by resolving isotopic overlaps for fragments c_5, c_{10}, c_{15}, and c_{20}. Lines of identity are represented by orange dashed lines. Spacer nucleotides that differ between the two gRNA are denoted in red. N(ms) denotes a phosphorothioate backbone at the 3'-end of the preceding 2'-O-methylribonucleotide.

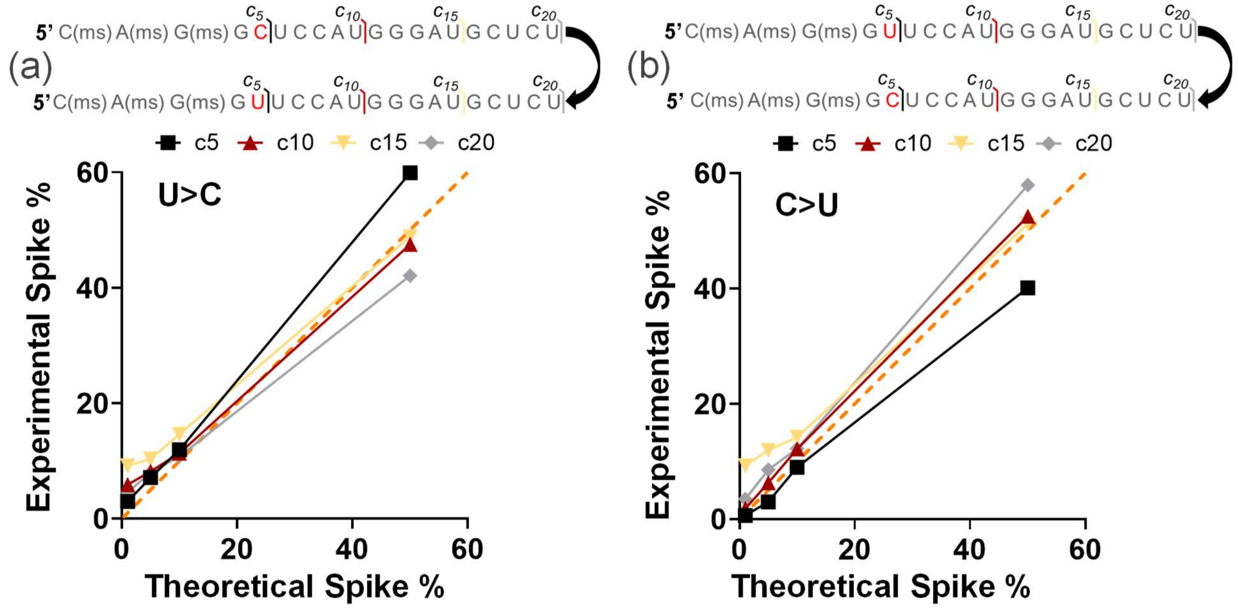

Figure S11. Top-down gRNA U>C base substitution localization and detection by isotopic fitting of *c*-ions generated across the entire spacer for mixtures of gRNA X spiked with 50%, 10%, 5% and 1% gRNA XD (C5 U>C). For sequence positions producing *c*-ions in multiple charge states, only percentages determined for the fragment in the charge state with highest cosine score are shown on the heatmaps. Isotopic fitting for gRNA X fragment ions in the absence of the U>C substitute are labelled as "0% U>C Spike". The first three nucleotides on the 5'-end feature 2'-O-methylation and phosphorothioate modifications.

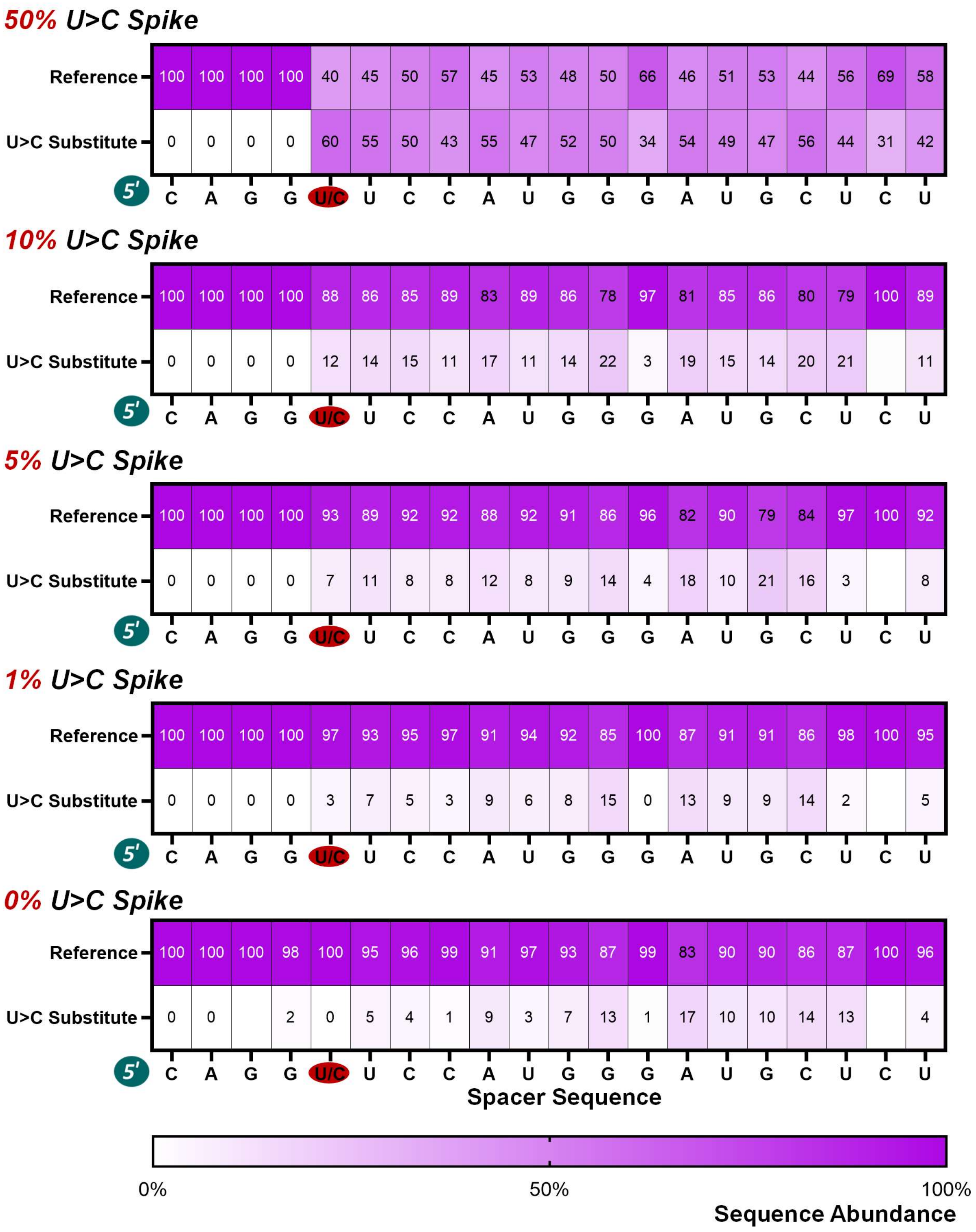
50% U>C Spike
Reference 100 100 100 100 40 45 50 57 45 53 48 50 66 46 51 53 44 56 69 58
U>C Substitute 0 0 0 0 60 55 50 43 55 47 52 50 34 54 49 47 56 44 31 42
5′ C A G G U/C U C C A U G G G A U G C U C U
10% U>C Spike
Reference 100 100 100 100 88 86 85 89 83 89 86 78 97 81 85 86 80 79 100 89
U>C Substitute 0 0 0 0 12 14 15 11 17 11 14 22 3 19 15 14 20 21 11
5′ C A G G U/C U C C A U G G G A U G C U C U
5% U>C Spike
Reference 100 100 100 100 93 89 92 92 88 92 91 86 96 82 90 79 84 97 100 92
U>C Substitute 0 0 0 0 7 11 8 8 12 8 9 14 4 18 10 21 16 3 8
5′ C A G G U/C U C C A U G G G A U G C U C U
1% U>C Spike
Reference 100 100 100 100 97 93 95 97 91 94 92 85 100 87 91 91 86 98 100 95
U>C Substitute 0 0 0 0 3 7 5 3 9 6 8 15 0 13 9 9 14 2 5
5′ C A G G U/C U C C A U G G G A U G C U C U
0% U>C Spike
Reference 100 100 100 98 100 95 96 99 91 97 93 87 99 83 90 90 86 87 100 96
U>C Substitute 0 0 2 0 5 4 1 9 3 7 13 1 17 10 10 14 13 4
5′ C A G G U/C U C C A U G G G A U G C U C U
Spacer Sequence
0%
50%
100%
Sequence Abundance

Figure S12. Top-down gRNA C>U base substitution localization and detection by isotopic fitting of *c*-ions generated across the entire spacer for mixtures of gRNA XD spiked with 50%, 10%, 5% and 1% gRNA X (representing C5 C>U). For sequence positions producing *c*-ions in multiple charge states, only percentages determined for the fragment in the charge state with highest cosine score are shown on the heatmaps. Isotopic fitting for gRNA XD fragment ions in the absence of the C>U substitute are labelled as "0% C>U Spike". The first three nucleotides on the 5'-end feature 2'-O-methylation and phosphorothioate modifications.

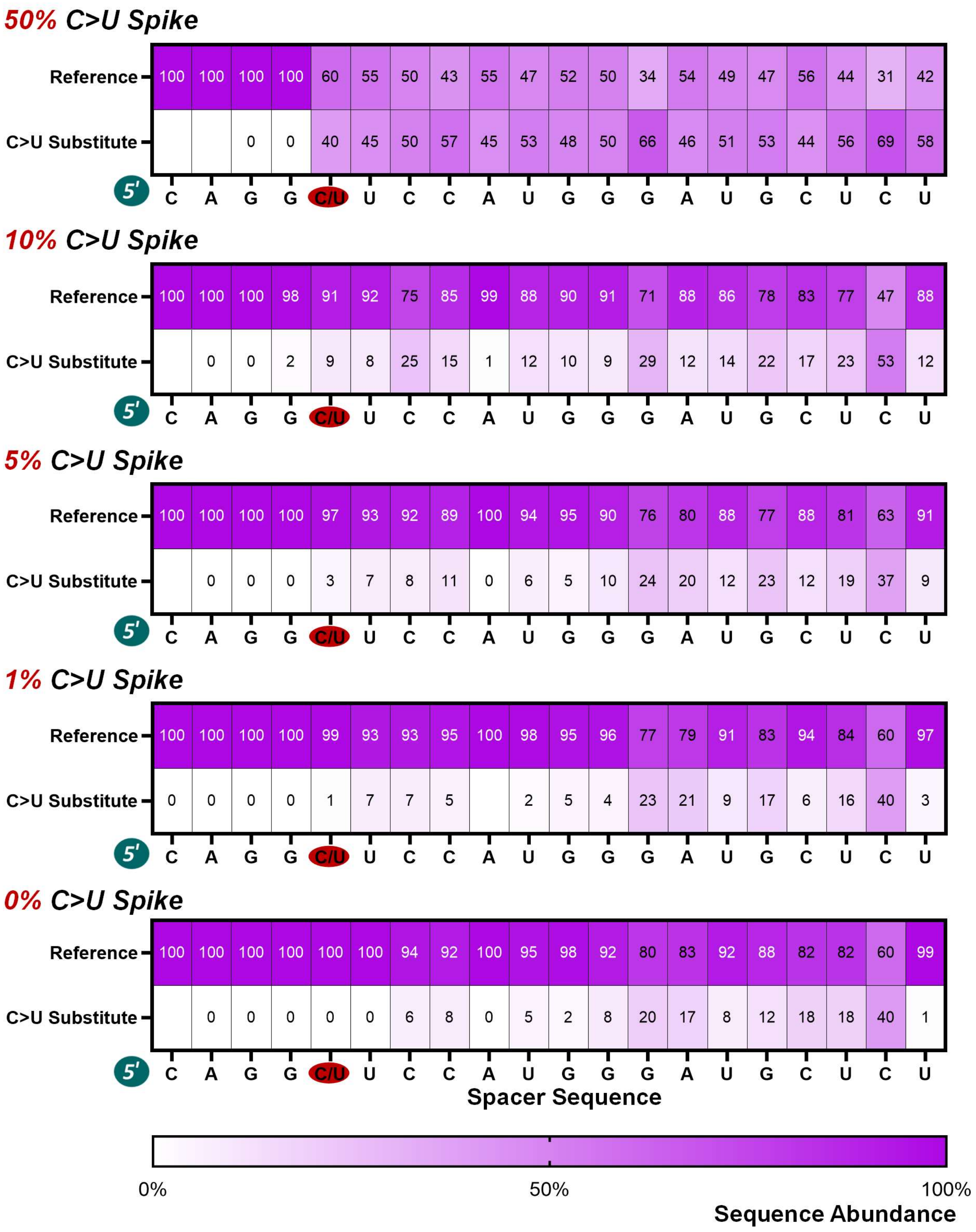
50% C>U Spike
Reference 100 100 100 100 60 55 50 43 55 47 52 50 34 54 49 47 56 44 31 42
C>U Substitute 0 0 40 45 50 57 45 53 48 50 66 46 51 53 44 56 69 58
5′ C A G G C/U U C C A U G G G A U G C U C U
10% C>U Spike
Reference 100 100 100 98 91 92 75 85 99 88 90 91 71 88 86 78 83 77 47 88
C>U Substitute 0 0 2 9 8 25 15 1 12 10 9 29 12 14 22 17 23 53 12
5′ C A G G C/U U C C A U G G G A U G C U C U
5% C>U Spike
Reference 100 100 100 100 97 93 92 89 100 94 95 90 76 80 88 77 88 81 63 91
C>U Substitute 0 0 0 3 7 8 11 0 6 5 10 24 20 12 23 12 19 37 9
5′ C A G G C/U U C C A U G G G A U G C U C U
1% C>U Spike
Reference 100 100 100 100 99 93 93 95 100 98 95 96 77 79 91 83 94 84 60 97
C>U Substitute 0 0 0 0 1 7 7 5 2 5 4 23 21 9 17 6 16 40 3
5′ C A G G C/U U C C A U G G G A U G C U C U
0% C>U Spike
Reference 100 100 100 100 100 100 94 92 100 95 98 92 80 83 92 88 82 82 60 99
C>U Substitute 0 0 0 0 0 6 8 0 5 2 8 20 17 8 12 18 18 40 1
5′ C A G G C/U U C C A U G G G A U G C U C U
Spacer Sequence
0%
50%
100%
Sequence Abundance

Table S9. Percentage of U>C substitutes and C>U substitutes detected at different concentrations averaging results from isotopic envelopes for fragments c_5 to c_{20}.

**Conc. %**	**c5:c20<br>U>C Substitution %**	**c5:c20<br>C>U Substitution %**
50%	48.1%	51.9%
10%	13.6%	17.0%
5%	9.8%	13.5%
1%	6.7%	11.0%
0%	6.9%	10.1%

Figure S13. Scores for gRNA Y sequence tags taken as a percent of gRNA Y and gRNA X sequence tag scores were plotted as a function of gRNA Y concentration to reveal a positive linear correlation. Line of identity is represented by orange dashed line. Spacer nucleotides that differ between the two gRNA are denoted in red. N(ms) denotes a phosphorothioate backbone at the 3'-end of the preceding 2'-O-methylribonucleotide.

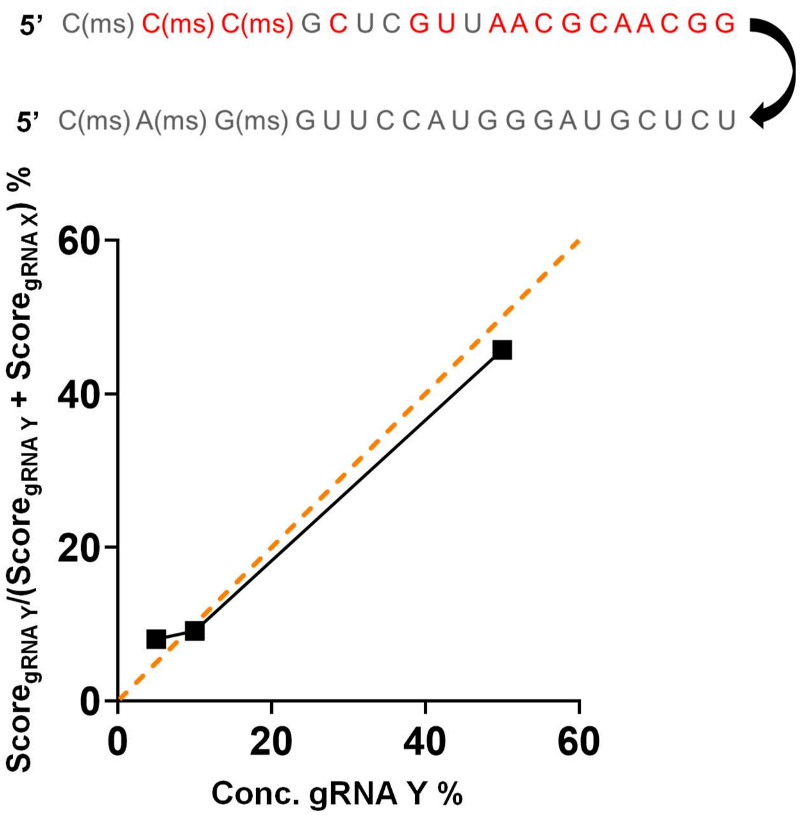

Figure S14. Scores for top-down *de novo* assignments of RNA modifications for the first 28 nucleotides of gRNA X, gRNA XA, gRNA XB, gRNA XC, gRNA XD, and gRNA Y. N and N(m) in the *x*-axis labels respectively indicate a ribonucleotide and a 2'-O-methylribonucleotide. N(s) and N(ms) in the *x*-axis labels denote a phosphorothioate backbone at the 3'-end of the preceding ribonucleotide and 2'-O-methylribonucleotide, respectively. * indicates that 2' O-methylation at G21 was instead assigned to U22 or U23. Assignments were limited to the first 27 nucleotides for gRNA XB.

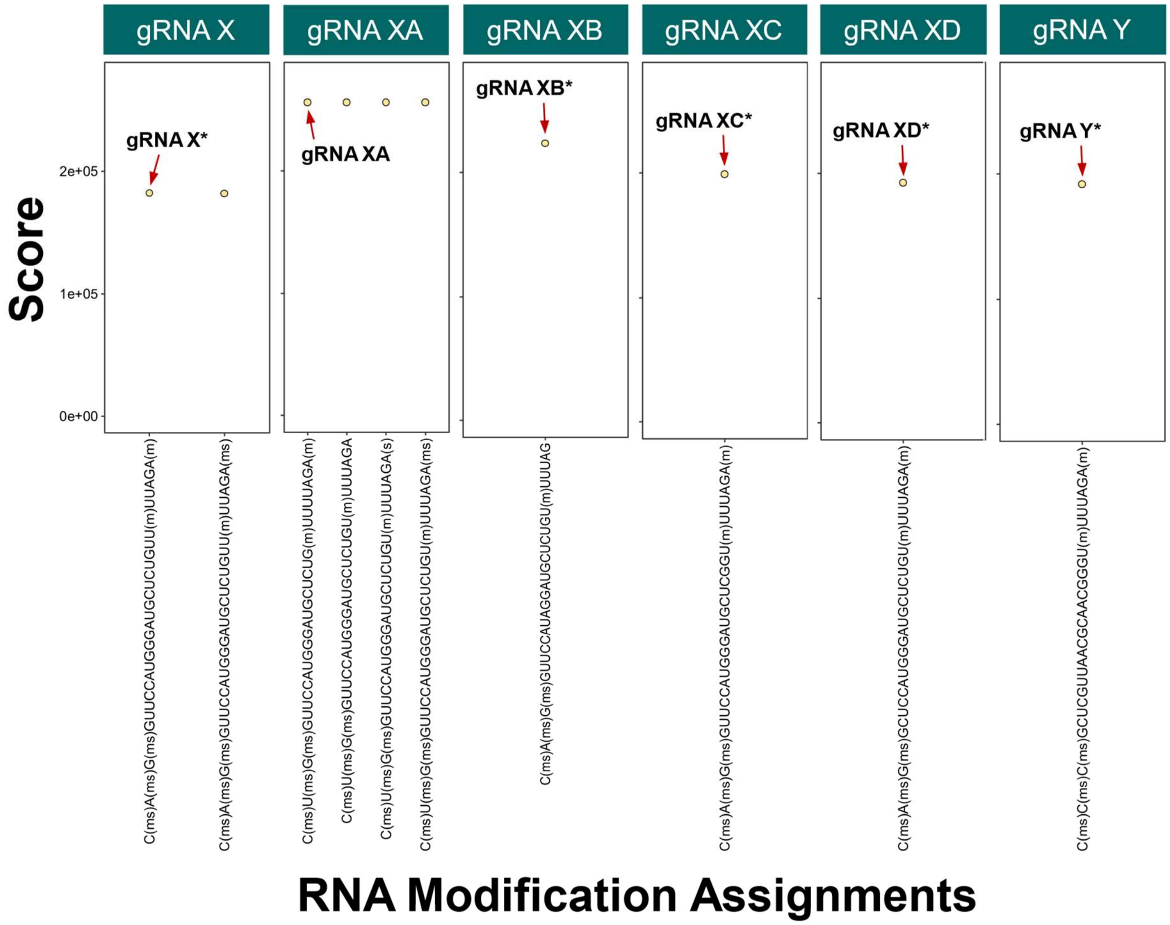

Supplement: Supplementary file 1 — oc3c00289_si_001.pdf [file oc3c00289_si_001.pdf]
